# Anthraquinones and Aloe Vera Extracts as Potential Modulators of Inflammaging Mechanisms: A Translational Approach from Autoimmune to Onco-Hematological Diseases

**DOI:** 10.3390/molecules30061251

**Published:** 2025-03-11

**Authors:** Raffaele Cordiano, Santino Caserta, Paola Lucia Minciullo, Alessandro Allegra, Sebastiano Gangemi

**Affiliations:** 1 Unit and School of Allergy and Clinical Immunology, Department of Clinical and Experimental Medicine, University of Messina, Via Consolare Valeria, 98125 Messina, Italy; raffaelecordiano@gmail.com (R.C.); sebastiano.gangemi@unime.it (S.G.); 2 Division of Hematology, Department of Human Pathology in Adulthood and Childhood “Gaetano Barresi”, University of Messina, Via Consolare Valeria, 98125 Messina, Italy; santino.caserta@polime.it (S.C.); aallegra@unime.it (A.A.)

**Keywords:** inflammaging, cytokine, autoimmunity, immune thrombocytopenia, hemolytic anemia, aloe vera, anthraquinones, aloe–emodin, aloin, aloesin

## Abstract

Inflammaging is a chronic, low-grade inflammatory state that contributes to age-related diseases, including cardiovascular disorders, osteoporosis, neurodegeneration, and cancer. This process involves immunosenescence, oxidative stress, and immune aging, all of which contribute to the breakdown of immune tolerance and the onset of autoimmune disorders. Aloe vera (AV) has recently gained attention for its immunomodulatory, anti-inflammatory, and antioxidant properties. This review explores the effects of AV extracts and anthraquinones (e.g., aloe–emodin, emodin, aloin) on key inflammaging-driven mechanisms in autoimmunity. Our analysis highlights AV’s ability to regulate hormone balance, autoantibody production, and cytokine/chemokine signaling (such as interleukin-1β, tumor necrosis factor-α, and interferon-γ). It modulates inflammatory pathways, including mitogen-activated protein kinases (MAPKs) and phosphatidylinositol 3-kinase/protein kinase B (PI3K/AKT), thereby inhibiting nuclear factor kappa-light-chain-enhancer of activated B-cell (NF-κB) activation. Additionally, AV enhances antioxidant defenses and restores immune balance by reducing Th1/Th17 subsets while promoting Th2-mediated regulation. Notably, AV also modulates inflammasome-mediated mechanisms and counteracts immunosenescence, which is driven by autophagy-related processes. These effects position AV as a potential integrative approach to mitigating inflammaging-driven autoimmunity. Furthermore, as inflammaging is increasingly recognized in onco-hematological diseases, AV-based strategies may offer novel therapeutic avenues. Future studies should focus on clinical validation, optimizing formulations, and expanding applications to broader age-related and immune-mediated disorders.

## 1. Introduction

The term inflammaging was first introduced by Franceschi et al. and refers to the integration of various molecular mechanisms that promote an enhanced pro-inflammatory state characteristic of the aging process [1]. Several contributing factors define inflammaging, including low-grade chronic inflammation, immunosenescence, self-debris accumulation, inflammasome activation, and an altered redox balance. These interconnected processes, resulting from the complex interplay between genetic predisposition and environmental influences, create a feedback loop that disrupts homeostasis in the human body [2,3]. When the body’s anti-inflammaging defenses—such as antioxidant mechanisms, DNA repair systems, programmed cell death, and autophagy—become overwhelmed, the mechanisms driving inflammaging contribute to the onset of age-related diseases, including cardiovascular disorders, osteoporosis, neurodegeneration, and cancer [4,5,6,7,8,9,10]. These processes—particularly immunosenescence—significantly impact the immune system, shaping what is referred to as immune-inflammaging [11,12,13]; compromises self-tolerance; and may influence the onset, progression, and severity of autoimmune diseases [14,15,16].

Autoimmunity arises when the immune system fails to distinguish between self and non-self, leading to tissue damage and the onset of autoimmune disorders [17]. These conditions affect millions of people worldwide, with a higher prevalence among women, posing significant health challenges. According to the 2019 Global Burden of Diseases, Injuries, and Risk Factors Study (GBD), there are approximately 67 million cases of autoimmune diseases globally [18]. Traditional treatment approaches rely primarily on immunosuppressive drugs that reduce the body’s immune response against self. While these therapies help manage symptoms, they do not address the underlying mechanisms driving autoimmunity [19].

Given the limitations of current therapies, research has shifted toward therapeutic strategies targeting modifiable factors to reduce the risk of autoimmune diseases. One auspicious approach involves nutritional supplementation, including the administration of vitamins, antioxidants, and micronutrients, which may play a pivotal role in managing autoimmune conditions [20,21,22,23]. Similarly, the emerging role of inflammaging has driven researchers to explore potential strategies to counteract this phenomenon. Some of these strategies overlap with those proposed for autoimmune diseases, such as caloric restriction, which can modulate key inflammatory pathways like NF-*κ*B, mTOR, and MAPK [24], as well as the supplementation of essential cofactors like zinc, which plays a crucial role in maintaining redox balance [25]. Other natural compounds, including resveratrol and flavonoids, have also gained attention due to their strong anti-inflammatory properties [26,27,28].

One of the most historically recognized sources of bioactive compounds is Aloe barbadensis Miller, commonly known as aloe vera (AV). This plant has been used for centuries in traditional medicine and has demonstrated a wide range of therapeutic properties, including skin protection, wound healing, and modulation of blood glucose and cholesterol levels, owing to the presence of various bioactive compounds such as anthraquinones, phenolic compounds, minerals, and vitamins [29].

With the increasing interest in plant-derived compounds as complementary therapies for various diseases, along with a growing focus on the mechanisms underlying aging-related disorders, this review aims to summarize key findings from the literature regarding the effects of AV extracts and their bioactive components, particularly anthraquinones, on the molecular pathways linking inflammaging and autoimmunity. We also provide a translational perspective on how this integrated therapy model can be applied to onco-hematologic diseases, potentially paving the way for new research directions in this field.

## 2. Search Strategy

We conducted a comprehensive PubMed search using the following keywords: “autoimmune” OR “autoimmunity” OR “rheumatoid arthritis” OR “lupus” OR “thyroiditis” OR “ankylosing spondylitis” OR “autoimmune diabetes” OR “anemia” OR “thrombocytopenia” AND “aloe vera” OR “anthraquinones” OR “emodin” OR “aloe-emodin” OR “aloin” OR “aloesin.” Our analysis included all research articles in the English language, applying no temporal or study-type restrictions, that explored the involvement of AV extract and/or the main anthraquinone compounds in autoimmune disorders.

## 3. Inflammaging Mechanisms and Autoimmunity

As previously mentioned, the term inflammaging involves various pro-inflammatory molecular mechanisms characterizing the aging process. This phenomenon arises from the intricate interplay between genetic predisposition and environmental influences. A state of low-grade chronic inflammation, enhanced immunosenescence, sustained activation of the inflammasome, and the accumulation of damaged macromolecules, organelles, and cells, together with a microenvironment where the redox balance is skewed towards pro-oxidant factors, represent the core components of inflammaging and the key players that interact to exert its detrimental effects [2,14]. External factors such as UV radiation, air pollution, smoking, temperature fluctuations, and infections caused by viruses, bacteria, and parasites significantly drive low-grade chronic inflammation [30,31]. Additionally, endogenous factors—including reactive oxygen species (ROS) and molecular changes such as epigenetic modifications and post-translational alterations—further push the cellular environment towards a pro-inflammatory state through the activation of Toll-like receptors (TLRs) and the inflammasome response mechanisms [32,33].

Oxidative stress plays a pivotal role in the intricate puzzle of inflammaging [34]. It is defined as an imbalance between antioxidants and pro-oxidants, with the latter predominating. The primary contributors to oxidative stress are ROS, such as the superoxide anion radical (O_2_^−^), hydroxyl radical (·OH), hydrogen peroxide (H_2_O_2_), and singlet oxygen (^1^O_2_), which are physiologically produced within cells, primarily in mitochondria, through enzymatic and oxidative processes [35]. However, when ROS production increases (e.g., due to infections, chronic stress, UV exposure, or pollutants) or when antioxidant defenses are diminished (e.g., reduced levels of glutathione peroxidase (GSX/Px), superoxide dismutase (SOD), catalase (CAT), or glutathione (GSH)), the resulting imbalance alters molecular signaling pathways and enzyme functions, leading to damage to nucleic acids, proteins, and lipids, and ultimately resulting in tissue damage [36,37].

Additionally, the generation and effects of ROS are closely linked to inflammation, another cornerstone of inflammaging. Oxidative stress is involved in the activation of various pro-inflammatory transcription factors, such as NF-κB and PI3K, and molecular pathways, including MAPK/ERK and the involvement of JNK and p38 proteins [38,39,40]. Furthermore, an interplay between ROS and the inflammasome mediated by nucleotide-binding domain-like receptor family pyrin domain-containing 3 (NLRP3), involved in many autoimmune and inflammatory diseases, along with the production of pro-inflammatory cytokines such as TNF-α, IL-1β, and IL-6, has been documented in organs targeted by oxidative stress [41,42,43,44].

The interaction between ROS and inflammation also underscores the active involvement of immune system cells. Factors that induce ROS production stimulate the release of pro-inflammatory cytokines and chemokines [45]. Similarly, immune system activation triggers specific molecular pathways involving pro-inflammatory cytokines and chemokines, leading to ROS production, as observed in macrophages and neutrophils [46,47,48].

The cells of our body naturally undergo an aging process known as immunosenescence, which plays a homeostatic and regulatory role, promoting the clearance of damaged and potentially cancerous cells. Immunosenescence synergizes with oxidative imbalance through two mechanisms: reduced cellular functions caused by oxidative damage to proteins, lipids, and carbohydrates, and cellular apoptosis due to the accumulation of detriments—effects that also impact the immune system, especially in an “oxi-inflammatory” microenvironment [3,49]. An accumulation of T-regulatory cells and an inverted CD4/CD8 ratio have been linked to immunosenescence, alongside specific alterations in lymphocyte subsets [50,51,52]. Excessive ROS production further pushes cells into a senescence-associated secretory phenotype (SASP), characterized by producing pro-inflammatory mediators and recruiting immune cells [53]. These mechanisms lead to inefficient phagocytosis and the accumulation of detriments acting as damage-associated molecular patterns (DAMPs), which trigger pro-inflammatory pathways. The increased burden on phagocytes to eliminate senescent cells leads to further ROS production, exacerbating oxidative imbalance and creating a vicious cycle [54].

These processes, driven by immunosenescence, low-grade chronic inflammation, and oxidative stress imbalance, also disrupt self-tolerance and can influence the onset, progression, and severity of autoimmune diseases [15,16] alongside a combination of other factors [55]. Bacterial and viral infections are known to trigger autoimmune responses and other environmental influences, including prolonged UV and chemical exposure, and certain lifestyle habits like smoking and alcohol consumption can initiate or worsen these conditions. Genetic predispositions also play a critical role, with specific human leukocyte antigen (HLA) alleles significantly increasing susceptibility to autoimmune diseases [56]. However, this predisposition alone is often insufficient to trigger the onset of the disease. The interplay between genetic factors and environmental influences can lead to epigenetic modifications, ultimately activating the latent susceptibility and transforming it into a fully developed autoimmune condition [57]. Additionally, hormonal dynamics contribute to the prevalence and onset of autoimmune diseases, as women are disproportionately affected [58]. Finally, the use of particular medications plays a crucial role in disease development and progression [17].

Impaired central tolerance, characterized by a reduced elimination of self-reactive T cells during early development, contributes to the formation of autoreactive T cells and an increased risk of autoimmune responses in aging [59,60]. Furthermore, T-cell receptor (TCR) signaling is altered in aging, leading to diminished accuracy, strength, and repertoire diversity of TCRs [61,62]. The reduced suppressive function of regulatory T cells (Tregs), mediated by transcription factors such as Foxp3, further drives the breakdown of immune tolerance and the development of autoimmunity. Evidence also points to functional alterations in B cells, natural killer (NK) cells, and myeloid lineage cells, including dendritic cells, macrophages, and monocytes [63]. Finally, low-grade chronic inflammation acts as an amplifier of autoimmune responses. This phenomenon occurs not only through an increase in pro-inflammatory cytokines such as IL-1, IL-6, and TNF-α but also through a reduction in anti-inflammatory defenses such as IL-10 [64,65].

## 4. AV: Overview, Therapeutic Uses, and Safety Profile

### 4.1. Generalities and Bioactive Compounds

Aloe barbadensis Miller, commonly known as AV, is a perennial succulent plant in the Liliaceae family, recognized for its high biological activity among over 500 species in its genus [66,67]. Originally from the southern Arabian Peninsula, it spread to regions like North Africa, Sudan, and others [68,69]. AV has been used as a healing herb since ancient Egyptian and Greek times and is still popular today in countries such as India, China, the West Indies, and Japan [70]. The plant’s leaves consist of three layers: the inner gel, rich in water, polysaccharides, vitamins, and amino acids; the middle latex layer, containing anthraquinones and other compounds; and the outer rind, which produces carbohydrates and proteins [71,72,73,74,75]. AV contains over 200 compounds, including bioactive substances like anthraquinones, phenolic compounds, enzymes, minerals, sugars, vitamins, lipids, hormones, proteins, and amino acids [76,77,78,79]. The anthraquinones, used historically as laxatives, are especially significant among the phenolic compounds, with their medicinal applications dating back over 4000 years [80]. As previously mentioned, anthraquinones are primarily located in the middle layer of the AV leaf structure, known as aloe latex. The main molecules include emodin, aloe–emodin, aloin (a mixture of aloin A/barbaloin and B/isobarbaloin), and aloesin.

**Figure 1:**
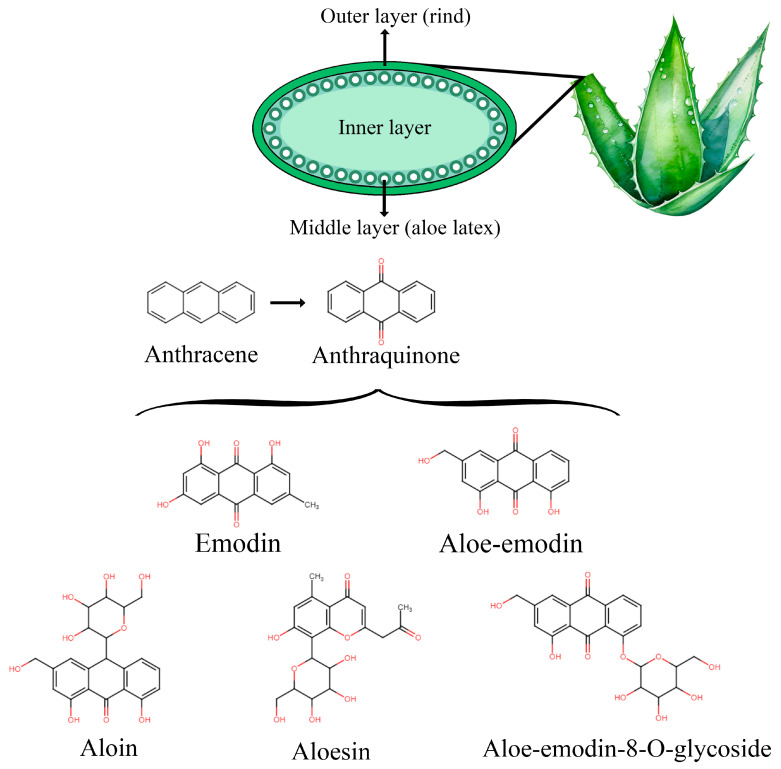
Representation of the structure of AV leaves and the main anthraquinone-derived compounds found in the aloe latex (middle layer).

The chemical structure of anthraquinones in AV is based on a core anthracene framework, consisting of three fused benzene rings (C_6_H_4_) that form an aromatic system. This structure is further characterized by the presence of two carbonyl groups (C=O) at positions 9 and 10 of the anthracene backbone. These carbonyl groups play a crucial role in the biological activity of anthraquinones, as they are involved in various chemical reactions. Anthraquinones in AV include compounds such as aloin, aloe–emodin, and emodin, each with slightly different substituents attached to the basic anthraquinone structure. Substituent groups on the anthraquinone skeleton, such as hydroxyl (-OH), methoxy (-OCH_3_), and methyl (-CH_3_), can significantly alter the compound’s properties, including its solubility, bioavailability, and potency. Additionally, these anthraquinones can exist in free form or as glycosides, where sugar molecules are attached to the anthraquinone structure. The glycoside form often has enhanced stability and may be more easily absorbed by the body. These structural features contribute to the diverse biological activities of anthraquinones, including their well-known laxative effects and antioxidant, anti-inflammatory, and antimicrobial properties. The molecular configuration of these compounds makes them highly reactive, enabling them to interact with cellular components and exert their therapeutic effects [81,82]. Figure 1 illustrates the structure of AV leaves along with the chemical structures of the main anthraquinones and their glycosides found in the middle layer.

The beneficial effects of the bioactive compound found in AV extract concern primarily their anti-inflammatory, antioxidant, and immunomodulatory activities, among others [83]. Compounds such as anthraquinones, bradykinase, lignin, aloctin, campesterol, and β-sitosterol are considered the main contributors to AV’s anti-inflammatory properties [68]. The inhibition of cyclooxygenase (COX) and lipoxygenase (LOX), key enzymes in the inflammatory response, has been highlighted in AV extracts [84]. Additionally, studies have documented the effects of aloin in reducing the production of ROS and the activation of the JAK1-STAT1/3 signaling pathway [85,86]. Similarly, aloe–emodin has been shown to inhibit pro-inflammatory cytokines such as TNF-α and IL-12, suppress nitric oxide production, downregulate iNOS expression, and interfere with pro-inflammatory pathways by reducing MAPK phosphorylation [87]. Promising immunomodulatory activity has also been attributed to AV. Specifically, AV has been shown to downregulate inflammasome-mediated responses, resulting in reduced expression of pro-IL-1β, NLRP3, caspase-1, IL-8, TNF-α, IL-6, and IL-1β, alongside decreased activation of signaling pathways such as NF-κB, p38, JNK, and ERK in human macrophages [88]. Finally, AV extract (AVH200^®^) has demonstrated the ability to reduce CD25 and CD3 expression on CD3^+^ T cells, suppress T-cell proliferation in a concentration-dependent manner, and decrease the levels of IL-2, IFN-γ, and IL-17A in human blood T cells derived from healthy individuals [89]. Regarding its antioxidant activity, the peel of AV, which contains numerous phenolic compounds, has been found to possess the highest antioxidant activity. It has been reported that vanillic acid can enhance the activities of SOD, CAT, and GSX/Px enzymes [90]. Moreover, anthraquinones and related compounds have demonstrated peroxyl radical scavenging activity and reducing capacity [91]. Finally, an increase in the plasma total antioxidant capacity was also reported in healthy volunteers after daily drinking of AV gel extract, further supporting the antioxidant potential of this plant [92].

### 4.2. Main Clinical Applications and Adverse Effects of AV

Over time, the medical applications of AV have expanded exponentially. The literature documents both human and animal studies highlighting its anti-diabetic effects. Evidence shows significant reductions in fasting and postprandial blood glucose levels, along with a decrease in HbA1c following oral AV extract administration in non-insulin-dependent diabetic patients [93,94]. Supporting its role in hyperglycemia management, another study on an animal model revealed improvements in obesity-induced insulin resistance [95]. The primary compound believed to play a pivotal role in addressing hyperglycemia is aloe-emodin-8-O-glycoside. This compound enhances glucose transport, uptake, and conversion to glycogen while regulating GLUT4 expression [96]. Additionally, aloe-emodin-8-O-glycoside has demonstrated anti-proliferative effects by modulating gene expression and inducing apoptosis [97]. Similarly, various anthraquinones have shown genotoxic and cytotoxic effects against multiple cancers, including chronic myeloid leukemia, breast cancer, colon cancer, and gastric cancer [98,99,100]. The anthracycline antibiotic class (e.g., doxorubicin, daunomycin) exemplifies the therapeutic potential of anthraquinones. These compounds are built on an anthraquinone-based fused tetracyclic ring system and represent an important family of antitumor agents widely used in chemotherapy [101]. In addition to their anticancer properties, there is evidence in the literature supporting the anti-inflammatory, anti-fungal, and anti-viral activities of anthraquinones [102,103,104,105]. Other specific peptides or proteins involved in stress tolerance and defense against pathogens have been identified in AV extracts, including saponins, polysaccharides, and pyrocatechol [106,107]. AV polysaccharides may also exhibit prebiotic properties by interacting with the human microbiota, potentially contributing to improved gastrointestinal health [108,109].

Anti-hyperlipidemic effects have been reported, including reductions in total cholesterol, triglycerides, LDL-C, and VLDL levels, along with increases in HDL levels [93,110], underscoring the potential of AV in managing lipid disorders. Moreover, hepatoprotective activity has been observed in liver damage models [111,112,113].

AV extract has also been widely explored for its applications in dermatology. Studies in the literature report its wound-healing effects [114,115], primarily mediated by acemannan and other glycoproteins [116]. Additionally, AV extracts have demonstrated anti-aging and moisturizing effects [117,118], largely attributed to the action of sterols [117]. The mechanisms underlying these effects include stimulation of cell proliferation and differentiation, promotion of wound contraction, epithelialization, and increased collagen density [119,120]. Moreover, AV helps counteract skin photoaging by reducing UVB-induced skin damage [118,121]. Beneficial effects have also been reported in inflammatory dermatological conditions such as psoriasis and acne vulgaris, although not all evidence is consistent [122,123,124,125].

Although AV provides numerous health benefits, assessing its safety and potential toxicity remains crucial. Studies on AV whole-leaf extract indicate significant dose-dependent toxicity. Acute toxicity trials reported an LD50 of 120.65 mg/kg in mice [126]. In a subchronic study, rats fed whole-leaf AV powder (2–8 g/kg for 90 days) showed increased defecation, reduced food efficiency, kidney weight changes, mesenteric lymph node proliferation, and renal pigment accumulation [127]. Hepatotoxicity has also been observed, with cases of toxic hepatitis linked to prolonged AV ingestion. Eight patients (six women, two men) developed toxic hepatitis after consuming AV for 3–260 weeks, with symptoms improving upon discontinuation [128,129].

The topical or oral use of the primary component—the inner gel layer—is generally considered safe. However, cases of generalized eczematous dermatitis and allergic contact dermatitis have been reported [129,130]. Cytotoxicity assays indicate that certain gel fractions disrupt intercellular junctions in fibroblast cultures [131]. Anthraquinones, found in AV latex, are the primary contributors to adverse AV effects. Their uncontrolled ingestion can lead to electrolyte imbalances, particularly hypokalemia, and cathartic colon, a condition characterized by a dilated and atonic colon due to chronic laxative use [132]. Long-term use has been linked to pseudomelanosis coli [133], diarrhea, abdominal pain, vomiting, and muscle weakness [129]. Further concerns involve gastrointestinal distress and potential carcinogenicity. Aloe–emodin exhibits genotoxicity in bacterial and mammalian cell assays [134,135]. Whole-AV extract induced large intestinal tumors in animal models, resembling human colorectal cancer in molecular pathways (MAPK, WNT, TGF-β) [136]. However, toxicity is significantly reduced in processed AV extracts: activated carbon filtration lowers anthraquinone levels from 8 mg/g to 0.08 mg/g [137]. Still, comparative studies suggest additional mutagenic factors persist post-processing [138].

AV also interacts with various drugs. Aloe–emodin inhibits cytochrome P450 enzymes (CYP3A4, CYP2D6), potentially altering drug metabolism. These interactions include enhanced corticosteroid absorption, exacerbation of digoxin- and diuretic-induced hypokalemia, and an increased bleeding risk with sevoflurane [68,139,140,141]. Despite concerns regarding certain AV components, commercial processing has led to safer formulations. International standards, such as those established by the International Aloe Science Council (IASC), limit aloin levels to below 10 ppm in oral AV products to mitigate genotoxic and carcinogenic risks [142]. However, while some AV-based beverages contain 5 ppm or less of aloin, safety concerns remain, particularly due to the variability in extract composition and the presence of other unknown derivatives [143].

Finally, despite strict regulations, the use of AV gel or latex remains contraindicated during pregnancy and breastfeeding and is considered potentially unsafe in children under 12 years of age and in individuals with chronic conditions [68]. Further studies are needed to define clear guidelines for safe AV consumption, dosage limits, and potential interactions with conventional drugs.

## 5. Effects of AV in Autoimmune Diseases

This section provides an overview of the studies identified in the literature based on our research. We have categorized the articles by disease into specific subsections, including a brief introduction to contextualize the pathophysiology, followed by the main findings of the reported studies. In the final subsection, we will further discuss the relevance of these findings in the context of inflammaging mechanisms, outlining the potential involvement of AV extracts and their compounds in autoimmune disorders.

### 5.1. Autoimmune Thyroiditis

Autoimmune thyroiditis represents the most common organ-specific autoimmune disease [144]. This group primarily includes Hashimoto’s thyroiditis (HT) and Graves’ disease (GD), which clinically result in hypothyroidism and hyperthyroidism, respectively. These conditions affect circulating levels of triiodothyronine (T3), thyroxine (T4), and thyroid-stimulating hormone (TSH), as well as the levels of autoantibodies such as thyroid peroxidase antibodies (TPOAb), thyroglobulin antibodies (TgAb), and thyroid-stimulating hormone receptor antibodies (TRAb). Despite their differing clinical manifestations, these diseases share common features, such as tissue damage (e.g., lymphocytic infiltration) and abnormal cytokine secretion [145]. Evidence in the literature links thyroid dysfunction to inflammation-related mechanisms [146]. For example, T4 has been shown to increase the production of ROS in immune cells [147], while the more potent T3 enhances metabolic activity and oxygen consumption, contributing to oxidative imbalance [148]. Additionally, T3 promotes the generation of M1 macrophages (pro-inflammatory phenotype), whereas low T3 levels increase the recruitment of inflammatory cells into tissues [149]. Other findings indicate that serum T3 stimulates the production of pro-inflammatory mediators (IL-1β, IL-6, TNF-α), while these cytokines, in turn, blunt the stimulatory effect of T3 by reducing peripheral thyroid hormone levels [150]. Despite these findings, the relationship between thyroid function and inflammaging remains unclear. A reduction in thyroid hormone levels during aging might be considered an adaptive mechanism to mitigate inflammaging and could play a protective role in immunosenescence [151].

The effects of AV extracts or anthraquinones have been evaluated in human and animal models of autoimmune thyroiditis. Below, we outline the main evidence supporting the potential role of these extracts/compounds in counteracting the effects of inflammaging in these pathologies.

Metro et al. [152] enrolled 45 women affected by Hashimoto’s thyroiditis (HT) with levothyroxine-untreated subclinical hypothyroidism, 30 of whom were treated orally with 50 mL/day of Aloe barbadensis Miller juice (ABMJ) for 9 months, and another group of 15 participants served as controls. Serum measurements showed significant improvement in TSH, free thyroxine (FT4), and TPOAb at months 3 and 9 (−61%, +23% and −56%). Meanwhile, free triiodothyronine (FT3) decreased significantly at 3 months (−16%) but showed no further reduction by the end of the study, resulting in a significant increase in the FT4:FT3 ratio (+33% at 3 months and +49% at 9 months).

A murine model of HT was evaluated by Sun et al. [153], in which the use of NaI was employed to induce experimental autoimmune thyroiditis in non-obese diabetic (NOD) mice. In this study, treatment with emodin at a concentration of 75 mg/kg for 3 days reduced TgAb levels in treated murine models compared to untreated ones. In addition, a reduction in CD3^+^CD4^+^IL-4^+^, CD3^+^CD4^+^IFN-γ^+^, CD3^+^CD8^+^IL-4^+^, and CD3^+^CD8^+^IFN-γ^+^ T cells in peripheral blood monocytes and splenic lymphocytes was found in HT mouse models treated with emodin, pointing to an immunoregulatory function between Th1 and Th2 populations. Finally, evidence that IFN-γ can increase HLA-II type expression in thyroid epithelial cells, hence triggering autoimmunity, supports a regulatory role of emodin in autoimmune processes.

Panda et al. [154] induced hyperthyroidism in murine models, dividing them into five groups: control, untreated hyperthyroid, two groups receiving 50 or 500 mg/kg/day of AV methanolic fraction (AVMF) orally, and a PTU-treated group (10 mg/kg/day, s.c.), all for 30 days. AVMF (50 mg/kg) and PTU significantly reduced T3, T4, and TSH levels while increasing antioxidant defenses (SOD, CAT, GPx, GSH) compared to untreated hyperthyroid rats. Elevated TNF-α and IL-6 levels in hyperthyroid animals decreased with AVMF (50 mg/kg) or PTU, alongside reduced TSH receptor (TSHR) expression. This model may resemble Graves’ thyroiditis, where TRAb-driven TSHR hyperactivation leads to excessive hormone secretion and thyroid hyperplasia, resulting in clinical hyperthyroidism [155].

### 5.2. Autoimmune Diabetes (AID)

AID is a potentially life-threatening metabolic disorder characterized by the progressive destruction of insulin-producing β-pancreatic cells by T lymphocytes, leading to impaired insulin production and hyperglycemia, two hallmark features of the disease [156]. Both autoimmune (type 1) and non-autoimmune (type 2) diabetes are associated with macro- and microvascular complications that can damage multiple organs and systems over time. Approximately 79,000 children under the age of 15 are estimated to develop AID annually worldwide. Similar to most autoimmune diseases, the etiology of AID is multifactorial, involving environmental, genetic, and immune factors [157]. In addition to T lymphocytes, other immune cells such as B lymphocytes, dendritic cells, macrophages, and natural killer (NK) cells are implicated in the pathogenesis, contributing to the development of insulitis [158]. A key mechanism underlying insulitis involves the chemotaxis of T lymphocytes [159,160]. Consequently, targeting chemokine receptors on leukocytes—such as CXCL4 expressed on T cells, including naïve T cells—could be a promising strategy for treating insulitis and preventing AID [161,162].

Studies have identified several chemokines associated with Th1 responses, such as CXCR3 and CCR5, as well as those linked to Th2 responses, including CCR3 and CCR4, as contributors to the onset and progression of AID [157]. Following this line of research, Shen et al. [163] investigated the effects of whole-plant extracts of Fallopia japonica, containing the anthraquinones emodin and physcion, in NOD murine models. The results demonstrated that treatment with the whole extract or emodin alone (4–40 mg/kg/day) for 30 weeks reduced diabetes incidence by over 70% in NOD mice. Histological analyses revealed delayed and reduced leukocyte infiltration into pancreatic islets in treated models. Furthermore, the study showed that emodin and physcion inhibited CXCR4-mediated migration in human T cells by suppressing MAPKs, ERK1/2, MAPKK, and MEK1/2.

An additional animal study [164] confirmed emodin’s inhibitory effect on CXCR4-mediated (IC50 = 0.3 µg/mL) and CCR5-mediated (IC50 = 0.75 µg/mL) migration in T cells. These results were attributed to the inhibitory effects on JNK- and p38-mediated pathways, which were directly linked to hydroxyl groups in anthraquinones. Finally, Malaguti et al. [165] demonstrated that diacerhein, an anthraquinone derivate known for its anti-inflammatory properties, reduced diabetes frequency in NOD mice in a dose-dependent manner (5 and 10 mg/kg/day) by downregulating pro-inflammatory cytokines such as IL-1β, TNF-α, and IFN-γ, known contributors to β-cell destruction in insulitis [166,167].

### 5.3. Multiple Sclerosis (MS)

MS is the most common autoimmune inflammatory disease affecting the central nervous system (CNS), characterized by the progressive loss of myelin proteins [168]. The disease affects over two million people worldwide and is most prevalent among young adults [169]. Demyelination of CNS cells leads to reduced sensitivity, difficulties with coordination and balance, muscle weakness, and visceral dysfunctions [170,171]. In the absence of effective treatments, the disease causes severe physical and psychological suffering for patients while also exerting a significant burden on families and society [172,173]. Environmental and genetic factors contribute to the onset of MS, which typically manifests as a relapsing–remitting disease, although some patients experience primary progressive MS without remission periods [174]. The activation of specific T lymphocyte subsets and their imbalance and associated cytokine production plays a crucial role in MS pathogenesis, promoting or suppressing neuroinflammation [175]. Specifically, TNF-α and IFN-γ, classic Th1-related pro-inflammatory cytokines, have been shown to contribute to tissue damage in MS [176]. Conversely, Th2 and Treg cells exert protective effects by suppressing immune responses and inhibiting excessive production of inflammatory mediators [177,178]. Th17 cells and related cytokines also play a significant role. An increase in IL-17-producing T cells has been observed in active MS lesions compared to inactive plaques. Moreover, IL-23 knockout mice were unable to generate the Th17 subset and were resistant to experimental autoimmune encephalomyelitis (EAE), the most common animal model for MS [179].

AV derivatives, particularly anthraquinones, have been evaluated in the EAE animal model. Alves et al. [180] demonstrated that intraperitoneal administration of 1 mg/kg/day for 7 days of O,O′-bis-(3′-iodopropyl)-1,4-dihydroxyanthraquinone, a derivative of mitoxantrone (an anthraquinone-derived antineoplastic agent structurally similar to doxorubicin), significantly improved clinical signs of the disease. This improvement was accompanied by a decrease in inflammatory cells, demyelination, and levels of IL-17, IFN-γ, IL-12p40, IL-6, TGF-β, CCL5, and CCL20 in the spinal cord, suggesting an immunoregulatory effect on lymphocyte populations and their chemotaxis. Similarly, Wei et al. [181] observed immunoregulatory effects in EAE models using oral administration of rhein (5 mg/kg/day) combined with the iridoid glycoside catalpol (40 mg/kg/day) for 40 days. The treatment reduced IL-2 and IL-17A levels while increasing IL-4 and IL-10 levels, the latter being a Treg-related anti-inflammatory cytokine. The authors also reported a reduced differentiation into Th1 cells, as indicated by lower T-bet levels, while promoting Th2 cell differentiation through increased GATA3 activity. Additionally, RORγt, a marker associated with Th17 differentiation, was downregulated, whereas Foxp3, crucial for Treg differentiation, was upregulated. Finally, neurological function scores significantly improved in treated animals compared to controls.

Microglial activation and inflammation have also been studied in EAE models. Zheng et al. [182] administered oral emodin at high (60 mg/kg/day) and low (30 mg/kg/day) doses in EAE murine models. The results demonstrated significant reductions in clinical scores and inflammatory cell infiltration in the spinal cord and brain, along with improved remyelination, as evidenced by increased expression of myelin-basic protein (MBP) and brain-derived neurotrophic factor (BDNF) in the high-emodin-treated group. Moreover, emodin significantly inhibited the phosphorylation of PI3K and AKT1, which are involved in the regulation of microglia inflammation [183], and reduced mRNA levels of IL-6, TGF-β, IL-17A, and RORγt, all of which are cytokines activating microglia. Additionally, Myd88 expression, a key mediator of TLR4 signaling pathways, was reduced in treated groups. Finally, Cui et al. [184] reported that emodin attenuated inflammation and demyelination in EAE models. Intraperitoneal administration of 20 mg/kg/day significantly reduced the levels of IL-1β, IL-6, IL-18, and TNF-α, as well as Iba-1, a marker associated with inflammation and microglial activation. Furthermore, the treatment downregulated SIRT1/PGC-1α expression and NLRP3-related molecules.

### 5.4. Rheumatoid Arthritis

Rheumatoid arthritis (RA) is a chronic autoimmune disease characterized by persistent inflammation in the joints, leading to pain, stiffness, swelling, and eventual joint damage. As a systemic condition, RA affects the joints and can also involve other organs, significantly impacting quality of life. Current therapeutic strategies for RA primarily focus on controlling inflammation and slowing disease progression, often through the use of nonsteroidal anti-inflammatory drugs (NSAIDs), disease-modifying antirheumatic drugs (DMARDs), and biologics. However, these treatments may be associated with adverse side effects, prompting patients and researchers to explore alternative therapies that could complement conventional treatments. AV, with its long history of use in traditional medicine, has gained attention in recent years for its potential therapeutic effects in managing RA symptoms. Ha et al. [185] supports the idea that AV can modulate immune responses. The study found that AV may exert an immune-regulatory effect by influencing the activity of T-cells, which play a central role in the autoimmune process underlying RA. By modulating immune responses, AV could help reduce the hyperactivation of the immune system that leads to joint inflammation and damage in RA. Additionally, AV’s antioxidant properties may help mitigate oxidative stress, which is known to contribute to the pathogenesis of RA. Clinical evidence also reinforces the potential benefits of AV in RA management. A randomized controlled trial [186] found that AV supplementation significantly improved disease activity and quality of life among RA patients. The study reported that patients who received AV exhibited reduced joint pain and swelling and better functional outcomes than those who did not receive the supplement. This clinical evidence suggests that AV could serve as a complementary therapy for managing RA symptoms and improving overall well-being. Furthermore, Hwang et al. [187] highlighted the potential of natural remedies, including AV, to alleviate the chronic pain and inflammation associated with RA. The authors emphasized the importance of exploring safe, natural alternatives that could enhance the effects of traditional RA therapies while minimizing side effects. Similarly, Kshirsagar et al. [188] explored the impact of AV on various inflammatory diseases, including RA, confirming its role in reducing inflammation and promoting healing.

### 5.5. Systemic Lupus Erythematosus

Systemic lupus erythematosus (SLE) is a chronic autoimmune disease that can cause widespread inflammation and damage to various organs, including the kidneys, skin, and joints. Renal involvement in SLE, known as lupus nephritis, is a common and serious complication that may lead to renal failure if not managed properly. In this regard, the study by Yuan et al. [189] investigates the potential therapeutic effects of emodin on renal injury in a mouse model of lupus. Their research demonstrated that emodin significantly reduced renal damage by modulating inflammatory pathways, particularly through the regulation of TNF-α (tumor necrosis factor-alpha) and ICAM-1 (intercellular adhesion molecule-1), both of which are key players in the inflammatory response and tissue damage in SLE.

The findings suggest that emodin may help mitigate renal injury by modulating immune responses and decreasing the infiltration of immune cells into the kidneys, offering a potential therapeutic approach for lupus nephritis. While further research is needed to confirm its efficacy in lupus management, emodin shows potential as a complementary therapy to traditional treatments by reducing inflammation and promoting immune balance.

### 5.6. Other Autoimmune Diseases

The literature provides limited reports on other autoimmune diseases, particularly regarding experimental autoimmune myocarditis (EAM) and ankylosing spondylitis (AS). Regarding EAM, this model mimics an inflammatory myocarditis condition, which is a major cause of dilated cardiomyopathy (DCM), potentially leading to heart failure and premature death in young adults [190,191]. Although its pathogenesis is not yet fully understood, there is evidence supporting the involvement of inflammatory and autoimmune factors [192]. In this experimental model, cytokines secreted by monocytes/macrophages and T cells are implicated in the induction and progression of EAM. Specifically, Th1-associated cytokines dominate the initial pro-inflammatory phase, while Th2 cytokines play a role in the recovery phase [193,194]. Song et al. [195] found that intragastric administration of emodin (50 mg/kg/day) in EAM mice significantly improved echocardiographic parameters, including LVEDd, LVEDs, LVFS, and the heart weight-to-body weight ratio, compared to controls. Histological analysis showed reduced inflammatory infiltrates in emodin-treated hearts, with fewer mononuclear cells, neutrophils, and multinucleated giant cells. Emodin also lowered myocardial NF-κB p65 protein levels, suggesting myocardial protection via NF-κB inhibition. Additionally, serum IL-1β and TNF-α levels were reduced, highlighting its systemic anti-inflammatory effects.

AS is a chronic autoimmune inflammatory disease that mainly affects the spine and sacroiliac joints (SIJs), along with soft tissues such as tendons and ligaments. Chronic inflammation leads to progressive fibrosis and calcification, which clinically manifests as back pain and increasing spinal rigidity [196]. Other joints, including the hips, shoulders, peripheral joints, and fingers/toes, may also be affected, along with extra-articular manifestations such as acute anterior uveitis and inflammatory bowel disease (IBD) [197]. In their in vitro study [198], Ma and colleagues analyzed the effects of emodin on fibroblasts derived from patients with AS. The results showed that emodin markedly reduced the viability of fibroblasts. In addition, the apoptotic rate of fibroblast cells was significantly increased, as indicated by the increase in apoptosis-related proteins Bax, active caspase-9, and caspase-3. In contrast, the expression of the anti-apoptotic protein Bcl-2 was significantly decreased. Finally, the authors found increased autophagic processes in fibroblasts through elevated expression of Beclin 1, Atg12, and Atg5.

The key features of the discussed studies are reported in Table 1.

### 5.7. Connecting the Dots: AV’s Influence on Inflammaging Mechanisms of Autoimmune Diseases

The cumulative evidence suggests that AV extracts may play a significant role in modulating autoimmune processes by addressing various inflammaging-related mechanisms.

In autoimmune thyroiditis, AV extracts influence critical pathways involved in thyroid function, such as regulating TSH receptor expression and reducing T3 and T4 levels in GD models [154]. Similarly, improvements in FT4 levels and FT4:FT3 ratio, and reductions in TSH, TPOAb, and TgAb levels in HT models, highlight the potential therapeutic effects of AV and its anthraquinone compounds, such as emodin [152,153]. Beyond its direct impact on thyroid function, AV also demonstrates immunomodulatory properties relevant to inflammaging mechanisms. Emodin has been shown to reduce thyroidal infiltration of T lymphocytes and modulate Th1 (IFN-γ-mediated) and Th2 (IL-4-mediated) responses, suggesting a role in restoring immune homeostasis [153]. Furthermore, AV exhibits potent anti-inflammatory and antioxidant effects, characterized by the downregulation of pro-inflammatory cytokines (TNF-α, IL-6) and the upregulation of antioxidant defenses, such as SOD, CAT, GPx enzymes, and GSH content [154]. These findings support the potential of AV-based interventions as integrative strategies for managing autoimmune thyroid disorders, targeting both endocrine and immune dysregulation.

Expanding upon these insights, anthraquinones also appear to regulate key pro-inflammatory cytokines involved in autoimmune diabetes, including IL-1β, TNF-α, and IFN-γ. Additionally, their modulation of CXCR4 and CCR5 signaling pathways through MAPKs (ERK1/2, JNK, and p38) further underscores their influence on inflammaging mechanisms [163,164]. These pathways are also implicated in oxidative stress-related inflammaging, including ROS generation induced by UV radiation and senescent cell activity [199,200].

In the context of MS and its experimental model (EAE), anthraquinones have been shown to exert neuroprotective effects by reducing pro-inflammatory cytokines and microglial activation [180,181,182,184]. Furthermore, they help restore immune balance by decreasing Th1 and Th17 responses, while promoting Th2 and Treg populations [181,182], leading to reduced demyelination and improved clinical outcomes [180,181,182]. At a molecular level, anthraquinones primarily target the PI3K/AKT1 and TLR4/MyD88 pathways, both of which drive NF-κB activation, a major regulator of inflammaging and age-related diseases [182,201,202]. Additionally, their effects on the SIRT1/PGC-1α axis and NLRP3 inflammasome suggest a broader impact on oxidative stress and immunosenescence processes [184].

Considering the anti-inflammatory, immunomodulatory, and antioxidant properties of AV, this plant extract emerges as a promising adjunctive therapy for rheumatoid arthritis (RA). Its ability to reduce inflammatory markers such as C-reactive protein, alleviate pain, and improve joint function highlights its potential as a natural treatment option for RA patients. While additional clinical studies are needed to fully validate its efficacy and safety, AV offers a complementary approach to standard RA therapies, potentially enhancing treatment outcomes and improving quality of life. Moreover, AV showed the capability to modulate the immune system by influencing the activity of T cells, fundamental in RA’s pathogenesis, and reducing the production of pro-inflammatory cytokines.

Intriguing evidence of emodin on renal injury derived from the research of Yuan and colleagues in a mouse model of lupus nephritis demonstrates that this molecule significantly reduced renal damage through the down-regulation of TNF-α (tumor necrosis factor-alpha) and ICAM-1 (intercellular adhesion molecule-1) and, consequently, led to a reduction in the infiltration of immune cells into the kidneys.

Beyond its effects on the disorders discussed above, emodin has been found to protect cardiac tissue by modulating inflammation via NF-κB inhibition [195]. This effect extends to systemic inflammation, where levels of NF-κB-associated cytokines—IL-1β and TNF-α—are significantly reduced following emodin treatment. Moreover, emodin’s potential role was highlighted in AS, particularly through its regulation of autophagy mechanisms [198]. Notably, autophagy declines with aging, exacerbating inflammaging by disrupting cellular homeostasis and activating NF-κB signaling [203,204], which contributes to a pro-inflammatory phenotype, either directly or through inflammasome activation. Furthermore, inflammatory signaling has been shown to suppress autophagy, creating a self-perpetuating cycle between autophagy inhibition and inflammasome activation [205]. For instance, TNF-α, when coupled with NF-κB activation, stimulates mTOR, a key inhibitor of autophagy. Conversely, in the absence of NF-κB signaling, TNF-α can promote Beclin 1 expression, an autophagy-enhancing factor. This response is further amplified by TNF-α-induced ROS production [206], thereby reinforcing the inflammaging process.

Below, we summarize the main evidence of AV’s effects on inflammaging mechanisms related to autoimmune diseases in a bullet-point format.

- *Modulate inflammatory signals influencing cytokine, chemokine, prostaglandin, and adhesion molecule production (↓ IL-1β* *, IL-2, IL-6, IL-8, IL-12, IL-17A, IL-18, TNF-α* *, IFN-γ* *, TGF-β* *, CXCR4, CXCR5, CCL5, CCL20, PGE2, and ICAM-1; ↑ IL-4 and IL-10);*
- *Exert an inhibitory influence on inflammatory MAPKs (ERK1/2, JNK, and p38), PI3K/AKT1, and TLR4/MyD88 molecular pathways;*
- *Reduce pro-inflammatory enzyme activity or mRNA expression (↓ COX-2, MMP-1, and MMP-13);*
- *Modulate angiogenesis (↓ VEGF);*
- *Regulate transcriptional factor expression linked with inflammation (↑ GATA3 and Foxp3; ↓ NF-κB, RORγt, and T-bet);*
- *Influence autoimmune key mechanisms reducing antibody production;*
- *Regulate immune response influencing T-cells subset population balance (Th1–Th2);*
- *Positively modulate autophagy (↑ Beclin1, Atg12, and Atg5 expression);*
- *Negative influence on inflammasome NLRP3 and related molecule activity;*
- *Reduced oxidative stress-enhancing anti-oxidant effectors (↑ SOD, GPx, GSH, and CAT activity).*

## 6. Future Perspectives: Autoimmune Cytopenias

### 6.1. Autoimmune Hemolytic Anemia

Autoimmune hemolytic anemia (AIHA) is an uncommon condition brought on by autoantibodies’ enhanced destruction of red blood cells (RBCs), either with or without complement activation. The direct antiglobulin test (DAT) or Coombs test can distinguish between two primary forms of AIHA based on the isotype and thermal properties of the autoantibody: cold agglutinin disease (CAD) and classic warm AIHA (wAIHA). While CAD is caused by an IgM that typically binds to erythrocytes at temperatures below 20 °C and significantly activates complement, wAIHA is maintained by an IgG that may weakly activate complement (particularly the IgG2 and IgG4 subclasses). The unusual forms (IgA or warm IgM driven, and DAT negative), the mixed forms (wAIHA with CAD), and the extremely uncommon paroxysmal cold hemoglobinuria are more uncommon. About half of cases of AIHA may be primary, or it may be linked to a number of conditions, including infections (parvovirus B19, mycoplasma pneumonia, mycobacterium tuberculosis, brucellosis, syphilis, EBV, CMV, hepatotropic virus, HIV, and SARS-CoV-2), autoimmune diseases (systemic lupus erythematosus, systemic sclerosis, vasculitis, Sjogren syndrome, antiphospholipid syndrome, autoimmune lymphoproliferative syndrome, autoimmune bowel disease, and autoimmune hepatitis), solid organ and hematopoietic stem cell transplants, a variety of medications (including immune checkpoint inhibitors anti-PD-1, PD-L1, and CTLA-4), congenital immunodeficiencies, and autoimmune hepatitis. Notably, infections, which are typically brought on by severe treatment immunosuppression or underlying immunodeficiency, can either be the cause of the disease’s genesis or a consequence of an already-existing AIHA. Clinically, the illness varies greatly, ranging from mild or compensated to severe or fatal cases. Blood loss, hypohaptoglobinemia, tissue necrosis or accelerated turnover, vitamin or iron deficiency, liver and renal disease, Gilbert syndrome, and many other conditions can cause changes in hemolytic indicators, such as unconjugated bilirubin, LDH, haptoglobin, and reticulocytes. The DAT is not entirely sensitive or specific, and a number of technical considerations are necessary for an accurate interpretation. Furthermore, therapy is primarily sponsored by expert consensus and has weak evidence support. Nonetheless, a number of novel medications have been studied or are being studied in prospective/controlled studies, providing fresh approaches to treating this illness. The diagnostic and treatment difficulties in AIHA, as well as the unmet needs for unusual types and relapsed/refractory cases, will all be covered in this review [207]. Large multiprotein complexes called inflammasomes are put together in cells in reaction to viral or environmental stress. Following inflammasome activation, caspase-1 starts pyroptotic cell death and cleaves the precursors of the inflammatory cytokines IL-1b and IL-18 [85]. It has been suggested that in AIHA there is an over-production of the pro-inflammatory cytokine IL-8, stimulated from IL-1 beta. In fact, Sanchez et al. investigated the anti-inflammatory properties of aloin in human oral epithelial cells and, since samples with high content in IL-1β stimulated IL-8 production in epithelial cells, they revealed that pretreatments with aloin inhibited IL-8 production by decreasing p38 and extracellular signal-regulated kinase pathway [66].

In this context, it is worth noting that in the field of hematology, the concept of inflammaging is increasingly being recognized. Although there are no studies in the literature specifically addressing the use of AV in autoimmune cytopenias, some indirect evidence suggests that derivatives from this plant may play a role in the pathogenesis of these diseases.

For example, CXCL10 (IP-10) is an important pro-inflammatory factor because it intensifies the effects of other pro-inflammatory cytokines, such as TNF-α, IL-6, L-1β, and CCL2. In detail, through controlling oxidative stress and inflammation, it plays a crucial role in the pathophysiology of AIHA. In different studies on liver diseases, it has been found that molecules from AV reduced the expression of inflammatory chemokines CCL2 and CXCL10 and reversed the expression levels of TNF-α, IL-6, IL-1β, and NF-κB in the liver tissues of NAFLD rats. In detail, different studies about NF-κB put in evidence that, with its pleiotropic regulatory role, it can bind to several promoters and have a role in controlling different inflammatory genes. Serum levels of TNF-α rise when cells undergo inflammatory changes, which triggers NF-κB activity and increases inflammatory responses. Activated NF-κB can then inversely enhance TNF-α production and result in cell/tissue injury [208].

### 6.2. Immune Thrombocytopenia

The acquired autoimmune disease known as immune thrombocytopenia (ITP) occurs when the body of the patient creates autoantibodies that target the platelets, causing them to be destroyed and reduced in quantity. Although the incidence of ITP varies by population and geographic location, there is no conclusive proof that there are notable variations around the globe [209]. ITP does not indicate a preference for any one race or ethnicity. According to epidemiological data, there are between 1.6 and 3.9 instances of ITP per 100,000 persons in Europe each year. Global data, where the incidence varies from 9.5 to 23.6 instances per 100,000 persons per year, cover a broader range. The 2011 PLATE study found that the annual incidence of ITP in Poland was 3.5 per 100,000 individuals. Although ITP can affect persons of any age or gender, incidence trends show that women between the ages of 30 and 60 are more likely to get it. The disease affects children between the ages of 1.9 and 6.4 out of 100,000 annually, with children under the age of 10 having the highest incidence. There is a higher chance of disease onset in men over 60 and in infancy. The generation and destruction of platelets are out of balance in ITP. The primary cause of thrombocytopenia is autoantibodies covering platelets, which makes it easier for macrophages’ Fcγ receptors to recognize them. Platelets are phagocytosed and destroyed as a result of this process, mostly in the liver and spleen. Antibodies can also prevent megakaryocytes in the bone marrow from doing their job, which lowers platelet production. Recent research has also demonstrated that T cells—both cytotoxic and regulatory—play a role in the pathophysiology of ITP. The maintenance of autoimmune reactions is specifically influenced by abnormalities in the function of regulatory T cells (Treg). Variable clinical manifestations result from the multiple etiology and varied pathophysiological mechanisms that underlie this complicated autoimmune illness. Understanding the course of the disease and improving treatment approaches depend on accurate classification based on comorbidities, symptom intensity, and length of illness. Severe clinical hurdles are presented by this illness, including a high risk of bleeding, resistance to treatment, and a severe impact on the patient’s quality of life. Long-term management and a customized strategy are necessary to address these problems, lower complications, and enhance therapeutic results. There are primary and secondary kinds of it. Understanding the differences between these kinds is essential to creating a successful treatment plan. The hallmark of primary ITP is thrombocytopenia with no known cause and no comorbidities. This type of thrombocytopenia is caused by autoantibodies that destroy platelets. Conversely, secondary ITP arises due to well-defined causes, including autoimmune disorders (like systemic lupus erythematosus), viral infections (like hepatitis C virus (HCV) and HIV), hematological malignancies (like lymphomas and leukemias), or medications (like heparin). Immune system dysregulation, which results in the generation of autoantibodies against platelet surface antigens and, ultimately, their death, is a crucial component of the pathophysiology of ITP, in both its main and secondary forms. A shorter platelet lifespan and greater thrombocytopenia are the results of this process, which include anomalies in the activation and regulation of B and T cells, cytokines, and macrophages [210]. Emodin has been shown to be able to reduce the secretion of TNF-α, IL-1β, and IL-6, and inhibit the expression of leukocyte chemokine CCL2. Moreover, other studies reported that aloe–emodin exerted anti-inflammatory effects by reducing the production of inflammatory factors (TNF-α and IL-6) through various pathways, such as NF-κB and PI3K/AKT [208]. AV has been shown to reduce the inflammatory process by lowering prostaglandin E2 production, cyclooxygenase pathway inhibition, and leukocyte adherence. According to Duansak, AV was found to reduce proinflammatory cytokines and leukocyte adhesion—which are strongly related to the pathogenesis of this cytopenia—which resulted in a considerable drop in TNF-α and IL6 levels. Additionally, reports have demonstrated that anthraquinones suppress the production of TNF-α, IL-6, IL-1β, MPO, MDA, CINC-1, MIP-2, ICAM-1, and MMP-9 in acute IBD and block the activation of NF-κB, JNK, p38 MAPK, Erk1/2, and 5-LOX. Given these facts, it has been demonstrated that natural anthraquinone derivatives have potential therapeutic effects in the prevention and/or treatment of a variety of inflammatory illnesses. In the gel, the intestinal flora converts aloin to reactive aloe–emodin, which lowers the DNA-binding activity of NF-Kβ and downregulates MMP-2/9, RAS, and vascular endothelial growth factor (VEGF) [211]. The PI3K/Akt signaling system, which is made up of a number of evolutionarily conserved enzymes that can be targeted with inhibitors of AKT, mTOR, PI3K, and associated components thereof, regulates cellular death, survival, inflammation, and proliferation. PI3K activation is nearly always brought on by cytokine, hormone, and growth factor signaling [212]. Recent research has demonstrated that PI3K/Akt signaling is a key regulator of inflammatory responses, and that blocking this pathway efficiently reduces the generation of inflammatory TNF-α and IL-6. Using a murine sepsis model, scientists evaluated the function of the PI3K/Akt/mTOR pathway and found that aloe emodin administration was adequate to suppress the upregulation of PI3K, Akt, and mTOR caused by LPS, suggesting that this chemical prevented the activation of PI3K signaling associated with sepsis. Endothelial cell-derived ROS can also induce PI3K/Akt pathway activation. Scientists found that aloe–emodin treatment suppressed both inflammatory cytokine production and oxidative stress in LPS-treated mice, while simultaneously suppressing PI3K pathway-related gene expression. As such, data suggested that aloe–emodin can attenuate inflammation and oxidative stress by inhibiting this PI3K/Akt/mTOR pathway. The association between aloe–emodin and the PI3K signaling pathway remains to be clarified in future studies. In summary, aloe–emodin has been identified as a potent anti-inflammatory and antioxidant compound that was able to protect against LPS-induced inflammation in our murine sepsis model at least in part via suppressing PI3K/Akt/mTOR signaling [209,213]. In autoimmune disorders, complement factors like C3a, C4a, and C5a are produced in large quantities when the complement system is first activated. These pieces cause inflammation, which leads to problems with the heart, lungs, brain, liver, kidneys, and other organs. Important pro-inflammatory proteins, including COX-2 and iNOS, are essential for the production of prostaglandin E2 (PGE2) and NO. In instances of inflammation, pro-inflammatory cytokines such as TNF-α, IL-1β, and IL-6 are markedly elevated. The effects of emodin on the production of inflammatory factors and serum antibodies in PBMCs induced by E. coli were investigated in this study. Emodin significantly reduced the production of pro-inflammatory proteins (iNOS and COX-2), inflammatory cytokines (IL-1β, TNF-α, and IL-6), and serum antibodies (C5 and C5a) in PBMCs that were triggered by E. coli. This implies that in the context of urosepsis, emodin can reduce inflammatory reactions and complement system activation. By enhancing the rise in the NAD/NADH ratio, AMPK can activate the SIRT1 deacetylase. Bei et al. showed that emodin can take role in the metabolism of fatty acids by altering the PPARα/γ-AMPK pathway. According to Song et al., amelogenin may be used to control the AMPK/SIRT1/NF-κB pathway, reducing brain damage brought on by sepsis. Western blot tests were performed to elucidate the expression levels of AMPK, p-AMPK, and SIRT1 in PBMCs in order to comprehend the emodin-based regulation of the AMPK/SIRT1 pathway. As a result, the addition of CC significantly reduced the synthesis of SIRT1 and p-AMPK. Following CC administration, increased levels of C5, C5a, COX-2, iNOS, IL-1β, TNF-α, and IL-6 were reported. CC also promoted the restoration of ROS and MDA levels while blocking the synthesis of SOD and GSH-Px. Thus, via regulating the AMPK/SIRT1 pathway in PBMCs, emodin helped to alleviate oxidative stress, inflammatory reactions, and complement system activation brought on by *E. coli*. An aberrant glomerular architecture, significantly enlarged renal capsules, evident necrotic tissue shedding, renal tubule blockages, and infiltration of inflammatory cells in the interstitium were reported in later studies employing rat urosepsis models. The renal tissue lesions in urosepsis rats considerably improved after emodin was administered. Notably, in urosepsis rats, emodin increased the synthesis of SOD and GSH-x while inhibiting the generation of ROS and MDA. In the meantime, emodin increased the expression of SIRT1 and p-AMPK while decreasing that of C5a, C5, IL-1β, TNF-α, IL-6, Cr, and BUN instead. Overall, emodin works through the AMPK/SIRT1 pathway to decrease oxidative stress, complementing system activation, inflammatory responses, and renal damage in rats with urosepsis [214]. LPS stimulation has been shown to increase the generation of ROS in macrophages, and ROS are secondary messengers that can control the activation of pro-inflammatory genes. Aloin’s antioxidant qualities have been demonstrated by earlier research. Aloin was found to reduce ROS accumulation in LPS-stimulated RAW264.7 cells in the current investigation. Moreover, ROS have the ability to strongly stimulate a number of signaling pathways, such as the JAK STAT and MAPK pathways. On the contrary, ROS inhibitor N acetyl L cysteine inhibited both the production of iNOS and the phosphorylation of JAK STATs. These findings lead us to speculate that aloin’s antioxidant activity against ROS in RAW264.7 cells may be the cause of its inhibitory effect on JAK1 STAT1/3. In conclusion, the study showed that aloin may partially reduce inflammation by preventing ROS-mediated activation of the JAK1 STAT1/3 signaling pathway in RAW264.7 macrophages [85].

## 7. Conclusions

AV and its bioactive compounds, particularly anthraquinones such as emodin, represent a promising natural therapeutic tool in the management of inflammaging-related mechanisms on autoimmune disorders. Scientific evidence highlights the ability of AV extracts to regulate immune responses, reduce inflammatory markers, and restore hormonal balance in conditions like rheumatoid arthritis, lupus nephritis, and autoimmune thyroiditis. In detail, research on emodin reveals its ability to reduce oxidative stress and modulate inflammatory pathways, particularly by regulating pro-inflammatory molecules such as TNF-α, IL-6, and IL-1β. These molecular actions play a crucial role in controlling the systemic inflammation seen in autoimmune diseases, thereby contributing to symptom management and disease progression. Further studies on lupus nephritis and thyroid disorders have demonstrated emodin’s efficacy in reducing renal injury and autoantibody levels, as well as improving organ function. These results suggest that AV may provide a holistic approach to managing autoimmune diseases by not only targeting inflammation but also restoring immune and endocrine balance, which are often disrupted in these conditions. However, while these findings are promising, further research is necessary to optimize the therapeutic use of AV.

Despite its potential benefits, the safety profile of AV remains a critical consideration. While the inner gel layer is generally recognized as safe, the presence of anthraquinones in whole-leaf extracts and latex raises concerns regarding toxicity. Adverse effects, including hepatotoxicity, nephrotoxicity, electrolyte imbalances, and gastrointestinal disturbances, have been reported in both experimental and clinical settings. Moreover, emerging evidence highlights the potential genotoxic and carcinogenic risks associated with chronic exposure to AV latex-derived anthraquinones, particularly aloe–emodin. These risks necessitate a careful evaluation of AV formulations, ensuring that products intended for therapeutic use adhere to strict processing standards to minimize harmful components.

While literature primarily supports the role of AV in immune modulation and inflammatory control, there is a notable gap in studies examining its potential impact on autoimmune hematological disorders, such as autoimmune anemia, thrombocytopenia, and neutropenia. In fact, the proposal to investigate AV’s therapeutic potential in these conditions is based on a sound scientific rationale rooted in its known immunomodulatory properties: in detail, AV has been demonstrated to downregulate inflammasome-mediated responses, leading to decreased activation of signaling pathways like NF-κB, p38, JNK, and ERK as well as decreased production of pro-IL-1β, NLRP3, TNF-α, IL-6, and IL-1β [81].

Autoimmune hematological disorders often involve complex dysregulation of the immune system that leads to the destruction or impairment of blood cells, including red blood cells, platelets, and neutrophils. Given AV’s ability to regulate immune responses, modulate inflammatory pathways, and improve organ function, it presents an intriguing candidate for addressing the underlying immune dysfunction that contributes to these hematological abnormalities. Moreover, it would be essential to assess the molecular mechanisms underlying these effects, particularly how AV compounds such as emodin interact with key immune pathways that govern hematopoiesis and immune tolerance.

In conclusion, while AV and its bioactive compounds, particularly emodin, have shown promise in managing autoimmune diseases such rheumatoid arthritis or systemic lupus erythematosus, additional research is crucial to expand their therapeutic potential into the realm of autoimmune hematological disorders. Furthermore, a deeper investigation into AV’s long-term safety profile is necessary to establish clear guidelines for its clinical application, ensuring that the benefits outweigh the risks associated with its consumption.

## Figures and Tables

**Table 1:** Characteristics of the studies evaluating the effects of AV extracts or anthraquinones in autoimmune diseases.

Authors	Study Type	Disease Models	AV Part(s) Used	Extraction Solvent(s) and Chromatographic Technique Used	Chemical Compound(s)/ Substance(s) Used (Dosage)	Main Biological or Chemical Marker Results	Cumulative Evidence
Metro et al. [152]	In vivo—women (30/15)	HT	AV leaf juice and pulp	-	Aloe barbadensis Miller juice (ABMJ) (50 mL/day)	↓ TSH (−61%), TPOAb (−56%) ↑ FT4 (+23%), FT4:FT3 ratio (+49%)	Regulate hormonal balance and antibody production
Sun et al. [153]	In vivo— murine	NaI-induced experimental autoimmune thyroiditis	-	-	Emodin (75 mg/kg)	↓ TgAb ↓ CD3+CD4+IL-4+/IFN-γ+ ↓ CD3CD8+ IL-4+/IFN-γ+	Regulate immune responses (Th1–Th2) Role in ↓ HLA-II type expression
Panda et al. [154]	In vivo— murine	Hypertiroidism (Graves’ disease model)	AV leaves	Petroleum, chloroform, methanol, and ethanol; LC-ESI-MS	AV methanolic fraction (AVMF) (50 and 500 mg/kg/day)	↓ IL-6, TNF-α ↑ SOD, CAT, GPx, GSH ↓ TSHR expression ↓ T3, T4	Anti-inflammatory and anti-oxidative effects Influence gene expression Regulate hormonal balance
Shen et al. [163]	In vivo—murine; in vitro	AID	None (root of *F. Japonica*)	Methanol; HPLC	Emodin (4–40 mg/kg/day)	↓ AID incidence >70% ↓ Leukocyte infiltration into pancreatic islets ↓ CXCR4-mediated migration via ERK1/2 and MEK1/2 inhibition	Influence lymphocyte activities and chemokine pathways
Shen et al. [164]	In vivo—murine; in vitro	AID	None (commercially available emodin product)	-	Emodin (IC50 = 0.3 µg/mL; 0.75 µg/mL)	↓ CXCR4- and CXCR5-mediated migration via JNK and p38 inhibition	Influence chemokine pathways
Malaguti et al. [165]	In vivo— murine	AID	-	-	Diacerhein (5 and 10 mg/kg/day)	↓ AID incidence ↓ IL-1β, TNF-α, and IFN-γ	Influence lymphocyte activities Anti-inflammatory effects
Alves et al. [180]	In vivo—murine; in vitro	EAE	None (commercially available mitoxantrone product)	Butanone; column chromatography	O,O′-bis-(3′-iodopropyl)-1,4-dihydroxyanthraquinone (1 mg/kg/day)	Improved clinical score ↓ Inflammatory cell infiltration ↓ Demyelination ↓ IL-17, IFN-γ, IL-12p40, IL-6, TGF-β, CCL5, and CCL20	Positive clinical impact Immunoregulatory effect on lymphocyte populations Influence lymphocyte chemotaxis Reduce demyelination Anti-inflammatory effects
Wei et al. [181]	In vivo—murine	EAE	-	-	Rhein (5 mg/kg/day) and Catalpol (40 mg/kg/day)	Improved clinical score ↓ IL-2 and IL-17A ↑ IL-4 and IL-10 ↓ T-bet and RORγt ↑ GATA3 and Foxp3	Positive clinical impact Anti-inflammatory effects Immunoregulatory effects on lymphocyte populations
Zheng et al. [182]	In vivo— murine	EAE	None (commercially available emodin product)	-	Emodin (30–60 mg/kg/day)	Improved clinical scores ↓ Inflammatory cell infiltration ↑ MBP and BDNF ↓ PI3K and AKT1 activation ↓ Mydd88 expression ↓ IL-6, TGF-β, IL-17A, and RORγt	Positive clinical impact Anti-inflammatory effects Improve remyelination
Cui et al. [184]	In vivo— murine; in vitro	EAE	None (commercially available emodin product)	-	Emodin (20 mg/kg/day)	↓ IL-1β, IL-6, IL-18, and TNF-α and Iba-1 ↓ SIRT1/PGC-and NLRP3-related molecules	Anti-inflammatory effects Modulate inflammasome response
Ha et al. [185]	In vitro (LPS-stimulated synoviocytes)	RA	-	-	Emodin (0, 1, or 10 ng/mL)	↓ IL-1β, IL-6, IL-18, and TNF-α ↓ PGE2, VEGF, MMP-1, and MMP-13 ↓ COX-2, MMP-1, and MMP-13 mRNA expression	Anti-inflammatory and anti-angiogenetic effects
Cheng et al. [186]	In vivo— mouse	RA	None (commercially available emodin product)	-	Emodin (10 mg/kg)	Relieves pain hypersensitivity of mice; ↓ spinal inflammation; inhibits spinal NLPR3 inflammasome activity; ↓ spinal AMPK expression	Positive clinical impact Anti-inflammatory effects
Kshirsagar et al. [188]	In vivo—mouse	RA	-	Dichloromethane	Aloe emodin (50 mg/kg); 4,5-dihydroxy-9,10-dioxo-9,10-dihydroanthracene-2 carboxylic acid (75 mg/kg)	↓ Paw edema; ↓ arthritic score	Positive clinical impact Anti-inflammatory effects
Yuan et al. [189]	In vivo—mouse	SLE	None (commercially available emodin product)	-	Emodin (0, 5, 10 and 20 mg/kg.d)	Down-regulation of TNF-α; ↓ level of ICAM-1	Theoretical base for a clinical application of emodin in the treatment of SLE
Song et al. [195]	In vivo—murine	EAM	None (commercially available Emodin product)	-	Emodin (50 mg/kg/day)	↑ Echocardiography parameters (LVEDs, LVEDs, LVFS) ↑ Hw/Bw ratio ↓ Inflammatory infiltrate ↓ NF-κB p65 expression ↓ IL-1β and TNF-α	Anti-inflammatory effects Cardioprotective effects
Ma et al. [198]	In vitro	AS	None (commercially available Emodin product)	-	Emodin (0, 2, 5, 10, or 20 µM)	↓ Viability ↑ apoptotic rate ↑ Bax, active caspase-9, and active caspase-3 ↓ Bcl-2 ↑ Beclin 1, Atg12, and Atg5	Induce autophagy and apoptosis in fibroblasts Reduced viability of fibroblasts

↑ = Increased; ↓ = decreased; AV = aloe vera; HT = Hashimoto thyroiditis; AID = autoimmune diabetes; AS = ankylosing spondylitis; COX-2 = cyclooxygenase-2; CAT = catalase; CCR5 = C-C chemokine receptor type 5; CXCR4 = C-X-C chemokine receptor type 4; DM = demyelination; EAE = experimental autoimmune encephalomyelitis; EAM = experimental autoimmune myocarditis; ELISA = enzyme-linked immunosorbent assay; FT3 = free triiodothyronine; FT4 = free thyroxine; GSH = glutathione; GPx = glutathione peroxidase; HIF-1α = hypoxia-inducible factor 1-alpha; HLA-II = human leukocyte antigen class II; ICAM-1 = intercellular adhesion molecule 1; IL = interleukin; JNK = c-Jun N-terminal kinase; LC-ESI-MS = liquid chromatography–electrospray ionization mass spectrometry; MAPK = mitogen-activated protein kinase; MMP = matrix metalloproteinase; MBP = myelin basic protein; NF-κB = nuclear factor kappa-light-chain-enhancer of activated B cells; NLRP3 = nucleotide-binding domain-like receptor family pyrin domain containing 3; PI3K = phosphoinositide 3-kinase; RA = rheumatoid arthritis; ROS = reactive oxygen species; SLE = systemic lupus erythematosus; SIRT1 = sirtuin 1; Th1 = T-helper 1 cells; Th2 = T-helper 2 cells; Th17 = T-helper 17 cells; TGF-β = transforming growth factor-beta; TLR4 = Toll-like receptor 4; TNF-α = tumor necrosis factor-alpha; TPOAb = thyroid peroxidase antibodies; TSH = thyroid-stimulating hormone; VEGF = vascular endothelial growth factor.

## References

[1] Franceschi C., Bonafè M., Valensin S., Olivieri F., De Luca M., Ottaviani E., De Benedictis G. (2000). Inflamm-Aging. An Evolutionary Perspective on Immunosenescence. Ann. N. Y. Acad. Sci..

[2] Franceschi C., Capri M., Garagnani P., Ostan R., Santoro A., Monti D., Salvioli S. (2019). Inflammaging. Handbook of Immunosenescence.

[3] Cannizzo E.S., Clement C.C., Sahu R., Follo C., Santambrogio L. (2011). Oxidative Stress, Inflamm-Aging and Immunosenescence. J. Proteom..

[4] Franceschi C., Capri M., Monti D., Giunta S., Olivieri F., Sevini F., Panourgia M.P., Invidia L., Celani L., Scurti M. (2007). Inflammaging and Anti-Inflammaging: A Systemic Perspective on Aging and Longevity Emerged from Studies in Humans. Mech. Ageing Dev..

[5] Fuentes E., Fuentes F., Vilahur G., Badimon L., Palomo I. (2013). Mechanisms of Chronic State of Inflammation as Mediators That Link Obese Adipose Tissue and Metabolic Syndrome. Mediat. Inflamm..

[6] Fulop T., Larbi A., Dupuis G., Le Page A., Frost E.H., Cohen A.A., Witkowski J.M., Franceschi C. (2017). Immunosenescence and Inflamm-Aging as Two Sides of the Same Coin: Friends or Foes?. Front. Immunol..

[7] Puspitasari Y.M., Ministrini S., Schwarz L., Karch C., Liberale L., Camici G.G. (2022). Modern Concepts in Cardiovascular Disease: Inflamm-Aging. Front. Cell Dev. Biol..

[8] Moyse E., Krantic S., Djellouli N., Roger S., Angoulvant D., Debacq C., Leroy V., Fougere B., Aidoud A. (2022). Neuroinflammation: A Possible Link between Chronic Vascular Disorders and Neurodegenerative Diseases. Front. Aging Neurosci..

[9] Olivieri F., Rippo M.R., Monsurrò V., Salvioli S., Capri M., Procopio A.D., Franceschi C. (2013). MicroRNAs Linking Inflamm-Aging, Cellular Senescence and Cancer. Ageing Res. Rev..

[10] Ginaldi L., Di Benedetto M.C., De Martinis M. (2005). Osteoporosis, Inflammation and Ageing. Immun. Ageing.

[11] Goronzy J.J., Weyand C.M. (2012). Immune Aging and Autoimmunity. Cell Mol. Life Sci..

[12] Vadasz Z., Haj T., Kessel A., Toubi E. (2013). Age-Related Autoimmunity. BMC Med..

[13] Wrona M.V., Ghosh R., Coll K., Chun C., Yousefzadeh M.J. (2024). The 3 I’s of Immunity and Aging: Immunosenescence, Inflammaging, and Immune Resilience. Front. Aging.

[14] Xia S., Zhang X., Zheng S., Khanabdali R., Kalionis B., Wu J., Wan W., Tai X. (2016). An Update on Inflamm-Aging: Mechanisms, Prevention, and Treatment. J. Immunol. Res..

[15] Weyand C.M., Fulbright J.W., Goronzy J.J. (2003). Immunosenescence, Autoimmunity, and Rheumatoid Arthritis. Exp. Gerontol..

[16] Montoya-Ortiz G. (2013). Immunosenescence, Aging, and Systemic Lupus Erythematous. Autoimmune Dis..

[17] Fu Y., Feng C., Qin S., Xing Z., Liu C., Liu Z., Yu H. (2024). Breaking Barriers: Advancing Cellular Therapies in Autoimmune Disease Management. Front. Immunol..

[18] GBD 2019 IMID Collaborators (2023). Global, Regional, and National Incidence of Six Major Immune-Mediated Inflammatory Diseases: Findings from the Global Burden of Disease Study 2019. EClinicalMedicine.

[19] Low C.E., Loke S., Chew N.S.M., Lee A.R.Y.B., Tay S.H. (2024). Vitamin, Antioxidant and Micronutrient Supplementation and the Risk of Developing Incident Autoimmune Diseases: A Systematic Review and Meta-Analysis. Front. Immunol..

[20] Harirchian M.H., Mohammadpour Z., Fatehi F., Firoozeh N., Bitarafan S. (2019). A Systematic Review and Meta-Analysis of Randomized Controlled Trials to Evaluating the Trend of Cytokines to Vitamin A Supplementation in Autoimmune Diseases. Clin. Nutr..

[21] Gioia C., Lucchino B., Tarsitano M.G., Iannuccelli C., Di Franco M. (2020). Dietary Habits and Nutrition in Rheumatoid Arthritis: Can Diet Influence Disease Development and Clinical Manifestations?. Nutrients.

[22] Stoiloudis P., Kesidou E., Bakirtzis C., Sintila S.-A., Konstantinidou N., Boziki M., Grigoriadis N. (2022). The Role of Diet and Interventions on Multiple Sclerosis: A Review. Nutrients.

[23] Balamurugan B.S., Marimuthu M.M.C., Sundaram V.A., Saravanan B., Chandrababu P., Chopra H., Malik T. (2024). Micro Nutrients as Immunomodulators in the Ageing Population: A Focus on Inflammation and Autoimmunity. Immun. Ageing.

[24] Chung K.W., Kim D.H., Park M.H., Choi Y.J., Kim N.D., Lee J., Yu B.P., Chung H.Y. (2013). Recent Advances in Calorie Restriction Research on Aging. Exp. Gerontol..

[25] Mocchegiani E., Costarelli L., Giacconi R., Cipriano C., Muti E., Tesei S., Malavolta M. (2006). Nutrient-Gene Interaction in Ageing and Successful Ageing. A Single Nutrient (Zinc) and Some Target Genes Related to Inflammatory/Immune Response. Mech. Ageing Dev..

[26] Marchal J., Pifferi F., Aujard F. (2013). Resveratrol in Mammals: Effects on Aging Biomarkers, Age-Related Diseases, and Life Span. Ann. N. Y. Acad. Sci..

[27] de la Lastra C.A., Villegas I. (2005). Resveratrol as an Anti-Inflammatory and Anti-Aging Agent: Mechanisms and Clinical Implications. Mol. Nutr. Food Res..

[28] Păcularu-Burada B., Cîrîc A.-I., Begea M. (2024). Anti-Aging Effects of Flavonoids from Plant Extracts. Foods.

[29] Gao Y., Kuok K.I., Jin Y., Wang R. (2019). Biomedical Applications of *Aloe vera*. Crit. Rev. Food Sci. Nutr..

[30] Baechle J.J., Chen N., Makhijani P., Winer S., Furman D., Winer D.A. (2023). Chronic Inflammation and the Hallmarks of Aging. Mol. Metab..

[31] Kornadt A.E., Kandler C. (2017). Genetic and Environmental Sources of Individual Differences in Views on Aging. Psychol. Aging.

[32] Gill R., Tsung A., Billiar T. (2010). Linking Oxidative Stress to Inflammation: Toll-like Receptors. Free Radic. Biol. Med..

[33] Martinon F. (2010). Signaling by ROS Drives Inflammasome Activation. Eur. J. Immunol..

[34] O’Rourke S.A., Shanley L.C., Dunne A. (2024). The Nrf2-HO-1 System and Inflammaging. Front. Immunol..

[35] Zorov D.B., Juhaszova M., Sollott S.J. (2014). Mitochondrial Reactive Oxygen Species (ROS) and ROS-Induced ROS Release. Physiol. Rev..

[36] Sies H., Berndt C., Jones D.P. (2017). Oxidative Stress. Annu. Rev. Biochem..

[37] Valko M., Leibfritz D., Moncol J., Cronin M.T.D., Mazur M., Telser J. (2007). Free Radicals and Antioxidants in Normal Physiological Functions and Human Disease. Int. J. Biochem. Cell Biol..

[38] Sun Y., Oberley L.W. (1996). Redox Regulation of Transcriptional Activators. Free Radic. Biol. Med..

[39] Thannickal V.J., Fanburg B.L. (2000). Reactive Oxygen Species in Cell Signaling. Am. J. Physiol. Lung Cell Mol. Physiol..

[40] Dröge W. (2002). Free Radicals in the Physiological Control of Cell Function. Physiol. Rev..

[41] Chen Y., Ye X., Escames G., Lei W., Zhang X., Li M., Jing T., Yao Y., Qiu Z., Wang Z. (2023). The NLRP3 Inflammasome: Contributions to Inflammation-Related Diseases. Cell Mol. Biol. Lett..

[42] Li Z., Guo J., Bi L. (2020). Role of the NLRP3 Inflammasome in Autoimmune Diseases. Biomed. Pharmacother..

[43] Hajam Y.A., Rani R., Ganie S.Y., Sheikh T.A., Javaid D., Qadri S.S., Pramodh S., Alsulimani A., Alkhanani M.F., Harakeh S. (2022). Oxidative Stress in Human Pathology and Aging: Molecular Mechanisms and Perspectives. Cells.

[44] Nordeng J., Schandiz H., Solheim S., Åkra S., Hoffman P., Roald B., Bendz B., Arnesen H., Helseth R., Seljeflot I. (2021). The Inflammasome Signaling Pathway Is Actively Regulated and Related to Myocardial Damage in Coronary Thrombi from Patients with STEMI. Mediat. Inflamm..

[45] Ryan K.A., Smith Jr M.F., Sanders M.K., Ernst P.B. (2004). Reactive Oxygen and Nitrogen Species Differentially Regulate Toll-like Receptor 4-Mediated Activation of NF-ΚB and Interleukin-8 Expression. Infect. Immun..

[46] Costa A.D.T., Garlid K.D. (2008). Intramitochondrial Signaling: Interactions among MitoKATP, PKCepsilon, ROS, and MPT. Am. J. Physiol. Heart Circ. Physiol..

[47] Fialkow L., Wang Y., Downey G.P. (2007). Reactive Oxygen and Nitrogen Species as Signaling Molecules Regulating Neutrophil Function. Free Radic. Biol. Med..

[48] Rendra E., Riabov V., Mossel D.M., Sevastyanova T., Harmsen M.C., Kzhyshkowska J. (2019). Reactive Oxygen Species (ROS) in Macrophage Activation and Function in Diabetes. Immunobiology.

[49] Gomez C.R., Nomellini V., Faunce D.E., Kovacs E.J. (2008). Innate Immunity and Aging. Exp. Gerontol..

[50] Feehan J., Tripodi N., Apostolopoulos V. (2021). The Twilight of the Immune System: The Impact of Immunosenescence in Aging. Maturitas.

[51] Rodrigues L.P., Teixeira V.R., Alencar-Silva T., Simonassi-Paiva B., Pereira R.W., Pogue R., Carvalho J.L. (2021). Hallmarks of Aging and Immunosenescence: Connecting the Dots. Cytokine Growth Factor Rev..

[52] Hashimoto K., Kouno T., Ikawa T., Hayatsu N., Miyajima Y., Yabukami H., Terooatea T., Sasaki T., Suzuki T., Valentine M. (2019). Single-Cell Transcriptomics Reveals Expansion of Cytotoxic CD4 T Cells in Supercentenarians. Proc. Natl. Acad. Sci. USA.

[53] Song S., Tchkonia T., Jiang J., Kirkland J.L., Sun Y. (2020). Targeting Senescent Cells for a Healthier Aging: Challenges and Opportunities. Adv. Sci..

[54] Martínez de Toda I., Ceprián N., Díaz-Del Cerro E., De la Fuente M. (2021). The Role of Immune Cells in Oxi-Inflamm-Aging. Cells.

[55] Yasmeen F., Pirzada R.H., Ahmad B., Choi B., Choi S. (2024). Understanding Autoimmunity: Mechanisms, Predisposing Factors, and Cytokine Therapies. Int. J. Mol. Sci..

[56] Pisetsky D.S. (2023). Pathogenesis of Autoimmune Disease. Nat. Rev. Nephrol..

[57] Mazzone R., Zwergel C., Artico M., Taurone S., Ralli M., Greco A., Mai A. (2019). The Emerging Role of Epigenetics in Human Autoimmune Disorders. Clin. Epigenet..

[58] Fairweather D., Beetler D.J., McCabe E.J., Lieberman S.M. (2024). Mechanisms Underlying Sex Differences in Autoimmunity. J. Clin. Investig..

[59] Thomas R., Wang W., Su D.-M. (2020). Contributions of Age-Related Thymic Involution to Immunosenescence and Inflammaging. Immun. Ageing.

[60] Zhao T.V., Sato Y., Goronzy J.J., Weyand C.M. (2022). T-Cell Aging-Associated Phenotypes in Autoimmune Disease. Front. Aging.

[61] Castro-Sanchez P., Teagle A.R., Prade S., Zamoyska R. (2020). Modulation of TCR Signaling by Tyrosine Phosphatases: From Autoimmunity to Immunotherapy. Front. Cell Dev. Biol..

[62] Oh J., Wang W., Thomas R., Su D.-M. (2017). Capacity of TTreg Generation Is Not Impaired in the Atrophied Thymus. PLoS Biol..

[63] Müller L., Di Benedetto S. (2023). From Aging to Long COVID: Exploring the Convergence of Immunosenescence, Inflammaging, and Autoimmunity. Front. Immunol..

[64] Pereira B.I., Akbar A.N. (2016). Convergence of Innate and Adaptive Immunity during Human Aging. Front. Immunol..

[65] Zuo L., Prather E.R., Stetskiv M., Garrison D.E., Meade J.R., Peace T.I., Zhou T. (2019). Inflammaging and Oxidative Stress in Human Diseases: From Molecular Mechanisms to Novel Treatments. Int. J. Mol. Sci..

[66] Sánchez M., González-Burgos E., Iglesias I., Gómez-Serranillos M.P. (2020). Pharmacological Update Properties of *Aloe vera* and Its Major Active Constituents. Molecules.

[67] Hossain M., MamunOrRashid A.N.M., Towfique N., Sen M. (2013). A Review on Ethnopharmacological Potential of *Aloe vera* L.. J. Intercult. Ethnopharmacol..

[68] Kaur S., Bains K. (2024). *Aloe barbadensis* Miller (*Aloe vera*). Int. J. Vitam. Nutr. Res..

[69] Kumar R., Singh A.K., Gupta A., Bishayee A., Pandey A.K. (2019). Therapeutic Potential of *Aloe vera*—A Miracle Gift of Nature. Phytomedicine.

[70] Rahman S., Carter P., Bhattarai N. (2017). *Aloe vera* for Tissue Engineering Applications. J. Funct. Biomater..

[71] Sahu P.K., Giri D.D., Singh R., Pandey P., Gupta S., Shrivastava A.K., Kumar A., Pandey K.D. (2013). Therapeutic and Medicinal Uses of *Aloe vera*: A Review. Pharmacol. Pharm..

[72] Surjushe A., Vasani R., Saple D.G. (2008). *Aloe vera*: A Short Review. Indian J. Dermatol..

[73] Lucini L., Pellizzoni M., Pellegrino R., Molinari G.P., Colla G. (2015). Phytochemical Constituents and in Vitro Radical Scavenging Activity of Different Aloe Species. Food Chem..

[74] Maan A.A., Nazir A., Khan M.K.I., Ahmad T., Zia R., Murid M., Abrar M. (2018). The Therapeutic Properties and Applications of *Aloe vera*: A Review. J. Herb. Med..

[75] Vogler B.K., Ernst E. (1999). *Aloe vera*: A Systematic Review of Its Clinical Effectiveness. Br. J. Gen. Pract..

[76] Mandal S.C., Nayak A.K., Dhara A.K. (2021). Herbal Biomolecules in Healthcare Applications.

[77] Fairbairn J.W. (1976). Biological Assay and Its Relation to Chemical Structure. Pharmacology.

[78] Godding E.W. (1976). Therapeutics of Laxative Agents with Special Reference to the Anthraquinones. Pharmacology.

[79] Malik E.M., Müller C.E. (2016). Anthraquinones as Pharmacological Tools and Drugs. Med. Res. Rev..

[80] Zhao L., Zheng L. (2023). A Review on Bioactive Anthraquinone and Derivatives as the Regulators for ROS. Molecules.

[81] Wu X., Ding W., Zhong J., Wan J., Xie Z. (2013). Simultaneous Qualitative and Quantitative Determination of Phenolic Compounds in *Aloe barbadensis* Mill by Liquid Chromatography-Mass Spectrometry-Ion Trap-Time-of-Flight and High Performance Liquid Chromatography-Diode Array Detector. J. Pharm. Biomed. Anal..

[82] Radha M.H., Laxmipriya N.P. (2015). Evaluation of Biological Properties and Clinical Effectiveness of *Aloe vera*: A Systematic Review. J. Tradit. Complement. Med..

[83] Catalano A., Ceramella J., Iacopetta D., Marra M., Conforti F., Lupi F.R., Gabriele D., Borges F., Sinicropi M.S. (2024). *Aloe vera*—An Extensive Review Focused on Recent Studies. Foods.

[84] Das S., Mishra B., Gill K., Ashraf M.S., Singh A.K., Sinha M., Sharma S., Xess I., Dalal K., Singh T.P. (2011). Isolation and Characterization of Novel Protein with Anti-Fungal and Anti-Inflammatory Properties from *Aloe vera* Leaf Gel. Int. J. Biol. Macromol..

[85] Ma Y., Tang T., Sheng L., Wang Z., Tao H., Zhang Q., Zhang Y., Qi Z. (2018). Aloin Suppresses Lipopolysaccharide-induced Inflammation by Inhibiting JAK1-STAT1/3 Activation and ROS Production in RAW264.7 Cells. Int. J. Mol. Med..

[86] Jiang K., Guo S., Yang C., Yang J., Chen Y., Shaukat A., Zhao G., Wu H., Deng G. (2018). Barbaloin Protects against Lipopolysaccharide (LPS)-Induced Acute Lung Injury by Inhibiting the ROS-Mediated PI3K/AKT/NF-ΚB Pathway. Int. Immunopharmacol..

[87] Li C.-Y., Suzuki K., Hung Y.-L., Yang M.-S., Yu C.-P., Lin S.-P., Hou Y.-C., Fang S.-H. (2017). Aloe Metabolites Prevent LPS-Induced Sepsis and Inflammatory Response by Inhibiting Mitogen-Activated Protein Kinase Activation. Am. J. Chin. Med..

[88] Budai M.M., Varga A., Milesz S., Tőzsér J., Benkő S. (2013). *Aloe vera* Downregulates LPS-Induced Inflammatory Cytokine Production and Expression of NLRP3 Inflammasome in Human Macrophages. Mol. Immunol..

[89] Ahluwalia B., Magnusson M.K., Isaksson S., Larsson F., Öhman L. (2016). Effects of *Aloe barbadensis* Mill. Extract (AVH200^®^) on Human Blood T Cell Activity in Vitro. J. Ethnopharmacol..

[90] Debnath T., Ghosh M., Lee Y.M., Nath N.C.D., Lee K.-G., Lim B.O. (2018). Identification of Phenolic Constituents and Antioxidant Activity of *Aloe barbadensis* Flower Extracts. Food Agric. Immunol..

[91] Sun Y.N., Li W., Lee S.H., Jang H.D., Ma J.Y., Kim Y.H. (2017). Antioxidant and Anti-Osteoporotic Effects of Anthraquinones and Related Constituents from the Aqueous Dissolved *Aloe* Exudates. Nat. Prod. Res..

[92] Prueksrisakul T., Chantarangsu S., Thunyakitpisal P. (2015). Effect of Daily Drinking of *Aloe vera* Gel Extract on Plasma Total Antioxidant Capacity and Oral Pathogenic Bacteria in Healthy Volunteer: A Short-Term Study. J. Complement. Integr. Med..

[93] Choudhary M., Kochhar A., Sangha J. (2014). Hypoglycemic and Hypolipidemic Effect of *Aloe vera* L. in Non-Insulin Dependent Diabetics. J. Food Sci. Technol..

[94] Dick W.R., Fletcher E.A., Shah S.A. (2016). Reduction of Fasting Blood Glucose and Hemoglobin A1c Using Oral *Aloe vera*: A Meta-Analysis. J. Altern. Complement. Med..

[95] Shin E., Shim K.-S., Kong H., Lee S., Shin S., Kwon J., Jo T.H., Park Y.-I., Lee C.-K., Kim K. (2011). Dietary Aloe Improves Insulin Sensitivity via the Suppression of Obesity-Induced Inflammation in Obese Mice. Immune Netw..

[96] Kumar R., Sharma B., Tomar N.R., Roy P., Gupta A.K., Kumar A. (2011). In Vivo Evaluation of Hypoglycemic Activity of Aloe Spp. and Identification of Its Mode of Action on GLUT-4 Gene Expression in Vitro. Appl. Biochem. Biotechnol..

[97] Kim H.J., Choi J.W., Ree J., Lim J.S., Lee J., Kim J.I., Thapa S.B., Sohng J.K., Park Y.I. (2022). Aloe Emodin 3-O-Glucoside Inhibits Cell Growth and Migration and Induces Apoptosis of Non-Small-Cell Lung Cancer Cells via Suppressing MEK/ERK and Akt Signalling Pathways. Life Sci..

[98] Manirakiza A., Irakoze L., Manirakiza S. (2021). Aloe and Its Effects on Cancer: A Narrative Literature Review. East Afr. Health Res. J..

[99] Chihara T., Shimpo K., Beppu H., Yamamoto N., Kaneko T., Wakamatsu K., Sonoda S. (2015). Effects of Aloe-Emodin and Emodin on Proliferation of the MKN45 Human Gastric Cancer Cell Line. Asian Pac. J. Cancer Prev..

[100] Suboj P., Babykutty S., Valiyaparambil Gopi D.R., Nair R.S., Srinivas P., Gopala S. (2012). Aloe Emodin Inhibits Colon Cancer Cell Migration/Angiogenesis by Downregulating MMP-2/9, RhoB and VEGF via Reduced DNA Binding Activity of NF-κB. Eur. J. Pharm. Sci..

[101] Selyutina O.Y., Mastova A.V., Polyakov N.E. (2023). The Interaction of Anthracycline Based Quinone-Chelators with Model Lipid Membranes: 1H NMR and MD Study. Membranes.

[102] Choi R.J., Ngoc T.M., Bae K., Cho H.-J., Kim D.-D., Chun J., Khan S., Kim Y.S. (2013). Anti-Inflammatory Properties of Anthraquinones and Their Relationship with the Regulation of P-Glycoprotein Function and Expression. Eur. J. Pharm. Sci..

[103] Agarwal S.K., Singh S.S., Verma S., Kumar S. (2000). Antifungal Activity of Anthraquinone Derivatives from Rheum Emodi. J. Ethnopharmacol..

[104] Barnard D.L., Fairbairn D.W., O’Neill K.L., Gage T.L., Sidwell R.W. (1995). Anti-Human Cytomegalovirus Activity and Toxicity of Sulfonated Anthraquinones and Anthraquinone Derivatives. Antivir. Res..

[105] Friedman M., Xu A., Lee R., Nguyen D.N., Phan T.A., Hamada S.M., Panchel R., Tam C.C., Kim J.H., Cheng L.W. (2020). The Inhibitory Activity of Anthraquinones against Pathogenic Protozoa, Bacteria, and Fungi and the Relationship to Structure. Molecules.

[106] Maan S.A., Faiesal A.A., Gamar G.M., El Dougdoug N.K. (2025). Efficacy of Bacteriophages with *Aloe vera* Extract in Formulated Cosmetics to Combat Multidrug-Resistant Bacteria in Skin Diseases. Sci. Rep..

[107] Kouser F., Kumar S., Bhat H.F., Hassoun A., Bekhit A.E.-D.A., Bhat Z.F. (2023). *Aloe barbadensis* Based Bioactive Edible Film Improved Lipid Stability and Microbial Quality of the Cheese. Foods.

[108] Gullón B., Gullón P., Tavaria F., Alonso J.L., Pintado M. (2015). In Vitro Assessment of the Prebiotic Potential of *Aloe vera* Mucilage and Its Impact on the Human Microbiota. Food Funct..

[109] Quezada M.P., Salinas C., Gotteland M., Cardemil L. (2017). Acemannan and Fructans from *Aloe vera* (*Aloe barbadensis* Miller) Plants as Novel Prebiotics. J. Agric. Food Chem..

[110] Moniruzzaman M., Rokeya B., Ahmed S., Bhowmik A., Khalil M.I., Gan S.H. (2012). In Vitro Antioxidant Effects of *Aloe barbadensis* Miller Extracts and the Potential Role of These Extracts as Antidiabetic and Antilipidemic Agents on Streptozotocin-Induced Type 2 Diabetic Model Rats. Molecules.

[111] Jain A., Jain S., Kothari N. (2022). An Experimental Study Performed to Compare the Hepatoprotective Activity of *Aloe vera* and Silymarin in Carbon Tetra Chloride (Ccl4)-Induced Hepatotoxicity in Albino Rabbits. Asian J. Pharm. Clin. Res..

[112] Cui Y., Ye Q., Wang H., Li Y., Yao W., Qian H. (2014). Hepatoprotective Potential of *Aloe vera* Polysaccharides against Chronic Alcohol-Induced Hepatotoxicity in Mice. J. Sci. Food Agric..

[113] Cui Y., Cheng Y., Guo Y., Xie Y., Yao W., Zhang W., Qian H. (2017). Evaluating the Hepatoprotective Efficacy of *Aloe vera* Polysaccharides against Subchronic Exposure of Aflatoxins B1. J. Taiwan Inst. Chem. Eng..

[114] Oryan A., Alemzadeh E., Mohammadi A.A., Moshiri A. (2019). Healing Potential of Injectable *Aloe vera* Hydrogel Loaded by Adipose-Derived Stem Cell in Skin Tissue-Engineering in a Rat Burn Wound Model. Cell Tissue Res..

[115] Takzaree N., Hadjiakhondi A., Hassanzadeh G., Rouini M.R., Manayi A., Zolbin M.M. (2016). Transforming Growth Factor-β (TGF-β) Activation in Cutaneous Wounds after Topical Application of *Aloe vera* Gel. Can. J. Physiol. Pharmacol..

[116] Sánchez-Machado D.I., López-Cervantes J., Sendón R., Sanches-Silva A. (2017). *Aloe vera*: Ancient Knowledge with New Frontiers. Trends Food Sci. Technol..

[117] Tanaka M., Misawa E., Yamauchi K., Abe F., Ishizaki C. (2015). Effects of Plant Sterols Derived from *Aloe vera* Gel on Human Dermal Fibroblasts in Vitro and on Skin Condition in Japanese Women. Clin. Cosmet. Investig. Dermatol..

[118] Saito M., Tanaka M., Misawa E., Yao R., Nabeshima K., Yamauchi K., Abe F., Yamamoto Y., Furukawa F. (2016). Oral Administration of *Aloe vera* Gel Powder Prevents UVB-Induced Decrease in Skin Elasticity via Suppression of Overexpression of MMPs in Hairless Mice. Biosci. Biotechnol. Biochem..

[119] Rahman M.S., Islam R., Rana M.M., Spitzhorn L.-S., Rahman M.S., Adjaye J., Asaduzzaman S.M. (2019). Characterization of Burn Wound Healing Gel Prepared from Human Amniotic Membrane and *Aloe vera* Extract. BMC Complement. Altern. Med..

[120] Teplicki E., Ma Q., Castillo D.E., Zarei M., Hustad A.P., Chen J., Li J. (2018). The Effects of *Aloe vera* on Wound Healing in Cell Proliferation, Migration, and Viability. Wounds.

[121] Misawa E., Tanaka M., Saito M., Nabeshima K., Yao R., Yamauchi K., Abe F., Yamamoto Y., Furukawa F. (2017). Protective Effects of Aloe Sterols against UVB-Induced Photoaging in Hairless Mice. Photodermatol. Photoimmunol. Photomed..

[122] Leng H., Pu L., Xu L., Shi X., Ji J., Chen K. (2018). Effects of Aloe Polysaccharide, a Polysaccharide Extracted from *Aloe vera*, on TNF-α-induced HaCaT Cell Proliferation and the Underlying Mechanism in Psoriasis. Mol. Med. Rep..

[123] Paulsen E., Korsholm L., Brandrup F. (2005). A Double-Blind, Placebo-Controlled Study of a Commercial *Aloe vera* Gel in the Treatment of Slight to Moderate Psoriasis Vulgaris. J. Eur. Acad. Dermatol. Venereol..

[124] Pal S., Raj M., Singh M., Saurav K., Paliwal C., Saha S., Sharma A.K., Singh M. (2024). The Effect of *Aloe vera* on Skin and Its Commensals: Contribution of Acemannan in Curing Acne Caused by Propionibacterium Acnes. Microorganisms.

[125] Miroddi M., Navarra M., Calapai F., Mancari F., Giofrè S.V., Gangemi S., Calapai G. (2015). Review of Clinical Pharmacology of *Aloe vera* L. in the Treatment of Psoriasis. Phytother. Res..

[126] Lagartoparra A. (2001). Comparative Study of the Assay of and the Estimate of the Medium Lethal Dose (LD50 Value) in Mice, to Determine Oral Acute Toxicity of Plant Extracts. Phytomedicine.

[127] Zhou Y., Feng Y., Wang H., Yang H. (2003). 90-Day Subchronic Toxicity Study of Aloe Whole-Leaf Powder. Wei Sheng Yan Jiu.

[128] Lee J., Lee M.S., Nam K.W. (2014). Acute Toxic Hepatitis Caused by an *Aloe vera* Preparation in a Young Patient: A Case Report with a Literature Review. Taehan Sohwagi Hakhoe Chi [Korean J. Gastroenterol.].

[129] Guo X., Mei N. (2016). *Aloe vera*: A Review of Toxicity and Adverse Clinical Effects. J. Environ. Sci. Health C Environ. Carcinog. Ecotoxicol. Rev..

[130] Ferreira M., Teixeira M., Silva E., Selores M. (2007). Allergic Contact Dermatitis to *Aloe vera*. Contact Dermat..

[131] Avila H., Rivero J., Herrera F., Fraile G. (1997). Cytotoxicity of a Low Molecular Weight Fraction from *Aloe vera* (*Aloe barbadensis* Miller) Gel. Toxicon.

[132] van Gorkom B.A., de Vries E.G., Karrenbeld A., Kleibeuker J.H. (1999). Review Article: Anthranoid Laxatives and Their Potential Carcinogenic Effects. Aliment. Pharmacol. Ther..

[133] Zhang R., Huang C., Wu F., Fang K., Jiang S., Zhao Y., Chen G., Dong R. (2023). Review on Melanosis Coli and Anthraquinone-Containing Traditional Chinese Herbs That Cause Melanosis Coli. Front. Pharmacol..

[134] Heidemann A., Völkner W., Mengs U. (1996). Genotoxicity of Aloeemodin in Vitro and in Vivo. Mutat. Res.-Rev. Genet. Toxicol..

[135] Hayes A.W., Pressman P., Clemens R., Singer A.W., Bauter M.R. (2024). Evaluation of 90-Day Repeated Dose Oral Toxicity of an *Aloe vera* Inner Leaf Gel Beverage. Food Chem. Toxicol..

[136] Boudreau M.D., Mellick P.W., Olson G.R., Felton R.P., Thorn B.T., Beland F.A. (2013). Clear Evidence of Carcinogenic Activity by a Whole-Leaf Extract of *Aloe barbadensis* Miller (*Aloe vera*) in F344/N Rats. Toxicol. Sci..

[137] Xia Q., Yin J.J., Fu P.P., Boudreau M.D. (2007). Photo-Irradiation of *Aloe vera* by UVA--Formation of Free Radicals, Singlet Oxygen, Superoxide, and Induction of Lipid Peroxidation. Toxicol. Lett..

[138] Guo X., Zhang S., Dial S.L., Boudreau M.D., Xia Q., Fu P.P., Levy D.D., Moore M.M., Mei N. (2014). In Vitro Investigation of the Mutagenic Potential of *Aloe vera* Extracts. Toxicol. Res..

[139] Davis R.H., Parker W.L., Murdoch D.P. (1991). *Aloe vera* as a Biologically Active Vehicle for Hydrocortisone Acetate. J. Am. Podiatr. Med. Assoc..

[140] Lee A., Chui P.T., Aun C.S.T., Gin T., Lau A.S.C. (2004). Possible Interaction between Sevoflurane and *Aloe vera*. Ann. Pharmacother..

[141] (2019). Abstracts of 48th ESCP Symposium on Clinical Pharmacy 23–25 October 2019, Ljubljana (Slovenia): The Digital Revolution Supporting Clinical Pharmacy through e-Health, Digital Support Systems, Big Data, and More. Int. J. Clin. Pharm..

[142] Pressman P., Clemens R., Hayes A.W. (2019). *Aloe vera* at the Frontier of Glycobiology and Integrative Medicine: Health Implications of an Ancient Plant. SAGE Open Med..

[143] Pressman P., Clemens R.A., Hayes A.W. (2022). EFSA Strikes Again: A Commentary on Flawed Analysis. Eur. J. Food Sci. Technol..

[144] Petranović Ovčariček P., Görges R., Giovanella L. (2024). Autoimmune Thyroid Diseases. Semin. Nucl. Med..

[145] Li Q., Wang B., Mu K., Zhang J.-A. (2019). The Pathogenesis of Thyroid Autoimmune Diseases: New T Lymphocytes—Cytokines Circuits beyond the Th1-Th2 Paradigm. J. Cell Physiol..

[146] Franceschi C., Ostan R., Mariotti S., Monti D., Vitale G. (2019). The Aging Thyroid: A Reappraisal within the Geroscience Integrated Perspective. Endocr. Rev..

[147] San Martín A., Griendling K.K. (2010). Redox Control of Vascular Smooth Muscle Migration. Antioxid. Redox Signal.

[148] Magsino Jr C.H., Hamouda W., Ghanim H., Browne R., Aljada A., Dandona P. (2000). Effect of Triiodothyronine on Reactive Oxygen Species Generation by Leukocytes, Indices of Oxidative Damage, and Antioxidant Reserve. Metabolism.

[149] Perrotta C., Buldorini M., Assi E., Cazzato D., De Palma C., Clementi E., Cervia D. (2014). The Thyroid Hormone Triiodothyronine Controls Macrophage Maturation and Functions: Protective Role during Inflammation. Am. J. Pathol..

[150] Rozing M.P., Westendorp R.G.J., Maier A.B., Wijsman C.A., Frölich M., de Craen A.J.M., van Heemst D. (2012). Serum Triiodothyronine Levels and Inflammatory Cytokine Production Capacity. Age.

[151] Ostan R., Bucci L., Capri M., Salvioli S., Scurti M., Pini E., Monti D., Franceschi C. (2008). Immunosenescence and Immunogenetics of Human Longevity. Neuroimmunomodulation.

[152] Metro D., Cernaro V., Papa M., Benvenga S. (2018). Marked Improvement of Thyroid Function and Autoimmunity by *Aloe barbadensis* Miller Juice in Patients with Subclinical Hypothyroidism. J. Clin. Transl. Endocrinol..

[153] Sun H., Ye Z., Li N., Jin F., Yan J., Wu K. (2018). Effect of Emodin on T Cell Subsets in NOD Mice with NaI-induced Experimental Autoimmune Thyroiditis. Mol. Med. Rep..

[154] Panda S., Sharma R., Khan A., Kar A. (2020). Ameliorative Effect of Aloe Gel against L-T4-Induced Hyperthyroidism via Suppression of Thyrotropin Receptors, Inflammation and Oxidative Stress. Mol. Biol. Rep..

[155] Akamizu T., Ikuyama S., Saji M., Kosugi S., Kozak C., McBride O.W., Kohn L.D. (1990). Cloning, Chromosomal Assignment, and Regulation of the Rat Thyrotropin Receptor: Expression of the Gene Is Regulated by Thyrotropin, Agents That Increase CAMP Levels, and Thyroid Autoantibodies. Proc. Natl. Acad. Sci. USA.

[156] Eizirik D.L., Colli M.L., Ortis F. (2009). The Role of Inflammation in Insulitis and Beta-Cell Loss in Type 1 Diabetes. Nat. Rev. Endocrinol..

[157] Chien S.-C., Wu Y.-C., Chen Z.-W., Yang W.-C. (2015). Naturally Occurring Anthraquinones: Chemistry and Therapeutic Potential in Autoimmune Diabetes. Evid.-Based Complement. Alternat Med..

[158] O’Reilly L.A., Hutchings P.R., Crocker P.R., Simpson E., Lund T., Kioussis D., Takei F., Baird J., Cooke A. (1991). Characterization of Pancreatic Islet Cell Infiltrates in NOD Mice: Effect of Cell Transfer and Transgene Expression. Eur. J. Immunol..

[159] Atkinson M.A., Wilson S.B. (2002). Fatal Attraction: Chemokines and Type 1 Diabetes. J. Clin. Investig..

[160] Bradley L.M., Asensio V.C., Schioetz L.K., Harbertson J., Krahl T., Patstone G., Woolf N., Campbell I.L., Sarvetnick N. (1999). Islet-Specific Th1, but Not Th2, Cells Secrete Multiple Chemokines and Promote Rapid Induction of Autoimmune Diabetes. J. Immunol..

[161] Meagher C., Sharif S., Hussain S., Cameron M.J., Arreaza G.A., Delovitch T.L. (2003). Cytokines and Chemokines in the Pathogenesis of Murine Type 1 Diabetes. Advances in Experimental Medicine and Biology.

[162] Bromley S.K., Mempel T.R., Luster A.D. (2008). Orchestrating the Orchestrators: Chemokines in Control of T Cell Traffic. Nat. Immunol..

[163] Shen M.-Y., Liu Y.-J., Don M.-J., Liu H.-Y., Chen Z.-W., Mettling C., Corbeau P., Chiang C.-K., Jang Y.-S., Li T.-H. (2011). Combined Phytochemistry and Chemotaxis Assays for Identification and Mechanistic Analysis of Anti-Inflammatory Phytochemicals in Fallopia Japonica. PLoS ONE.

[164] Shen M.-Y., Lin Y.-P., Yang B.-C., Jang Y.-S., Chiang C.-K., Mettling C., Chen Z.-W., Sheu J.-R., Chang C.L., Lin Y.-L. (2012). Catenarin Prevents Type 1 Diabetes in Nonobese Diabetic Mice via Inhibition of Leukocyte Migration Involving the MEK6/P38 and MEK7/JNK Pathways. Evid.-Based Complement. Alternat. Med..

[165] Malaguti C., Vilella C.A., Vieira K.P., Souza G.H.M.F., Hyslop S., Zollner R. (2008). de L. Diacerhein Downregulate Proinflammatory Cytokines Expression and Decrease the Autoimmune Diabetes Frequency in Nonobese Diabetic (NOD) Mice. Int. Immunopharmacol..

[166] Rabinovitch A. (1998). An Update on Cytokines in the Pathogenesis of Insulin-Dependent Diabetes Mellitus. Diabetes Metab. Rev..

[167] Suk K., Kim S., Kim Y.H., Kim K.A., Chang I., Yagita H., Shong M., Lee M.S. (2001). IFN-Gamma/TNF-Alpha Synergism as the Final Effector in Autoimmune Diabetes: A Key Role for STAT1/IFN Regulatory Factor-1 Pathway in Pancreatic Beta Cell Death. J. Immunol..

[168] Pugliatti M., Sotgiu S., Rosati G. (2002). The Worldwide Prevalence of Multiple Sclerosis. Clin. Neurol. Neurosurg..

[169] Browne P., Chandraratna D., Angood C., Tremlett H., Baker C., Taylor B.V., Thompson A.J. (2014). Atlas of Multiple Sclerosis 2013: A Growing Global Problem with Widespread Inequity. Neurology.

[170] Preziosi G., Gordon-Dixon A., Emmanuel A. (2018). Neurogenic Bowel Dysfunction in Patients with Multiple Sclerosis: Prevalence, Impact, and Management Strategies. Degener. Neurol. Neuromuscul. Dis..

[171] Aharony S.M., Lam O., Corcos J. (2017). Evaluation of Lower Urinary Tract Symptoms in Multiple Sclerosis Patients: Review of the Literature and Current Guidelines. J. Assoc. Urol. Can. [Can. Urol. Assoc. J.].

[172] Magyari M., Sorensen P.S. (2019). The Changing Course of Multiple Sclerosis: Rising Incidence, Change in Geographic Distribution, Disease Course, and Prognosis. Curr. Opin. Neurol..

[173] Berglund R., Guerreiro-Cacais A.O., Adzemovic M.Z., Zeitelhofer M., Lund H., Ewing E., Ruhrmann S., Nutma E., Parsa R., Thessen-Hedreul M. (2020). Microglial Autophagy-Associated Phagocytosis Is Essential for Recovery from Neuroinflammation. Sci. Immunol..

[174] Lassmann H., van Horssen J. (2011). The Molecular Basis of Neurodegeneration in Multiple Sclerosis. FEBS Lett..

[175] Steinman L. (2007). A Brief History of T(H)17, the First Major Revision in the T(H)1/T(H)2 Hypothesis of T Cell-Mediated Tissue Damage. Nat. Med..

[176] Kallaur A.P., Oliveira S.R., Simão A.N.C., Alfieri D.F., Flauzino T., Lopes J., de Carvalho Jennings Pereira W.L., de Meleck Proença C., Borelli S.D., Kaimen-Maciel D.R. (2017). Cytokine Profile in Patients with Progressive Multiple Sclerosis and Its Association with Disease Progression and Disability. Mol. Neurobiol..

[177] Oreja-Guevara C., Ramos-Cejudo J., Aroeira L.S., Chamorro B., Diez-Tejedor E. (2012). TH1/TH2 Cytokine Profile in Relapsing-Remitting Multiple Sclerosis Patients Treated with Glatiramer Acetate or Natalizumab. BMC Neurol..

[178] Sakaguchi S., Yamaguchi T., Nomura T., Ono M. (2008). Regulatory T Cells and Immune Tolerance. Cell.

[179] Moser T., Akgün K., Proschmann U., Sellner J., Ziemssen T. (2020). The Role of TH17 Cells in Multiple Sclerosis: Therapeutic Implications. Autoimmun. Rev..

[180] Alves C.C.S., Castro S.B.R., Costa C.F., Dias A.T., Alves C.J., Rodrigues M.F., Teixeira H.C., Almeida M.V., Ferreira A.P. (2012). Anthraquinone Derivative O,O′-Bis-(3′-Iodopropyl)-1,4-Dihydroxyanthraquinone Modulates Immune Response and Improves Experimental Autoimmune Encephalomyelitis. Int. Immunopharmacol..

[181] Wei M., Yang T., Li Q., Zhou D., Du Z., Fan Y. (2019). Protective Effects of Catalpol and Rhein in Murine Experimental Autoimmune Encephalomyelitis via Regulation of T Helper (Th) 1, Th2, Th17, and Regulatory T Cell Responses. Chung Chih Ying Wen Pan [J. Tradit. Chin. Med.].

[182] Zheng K., Lv B., Wu L., Wang C., Xu H., Li X., Wu Z., Zhao Y., Zheng Z. (2022). Protecting Effect of Emodin in Experimental Autoimmune Encephalomyelitis Mice by Inhibiting Microglia Activation and Inflammation via Myd88/PI3K/Akt/NF-ΚB Signalling Pathway. Bioengineered.

[183] da Silva L.C., Lima I.V.d.A., da Silva M.C.M., Corrêa T.A., de Souza V.P., de Almeida M.V., de Oliveira A.C.P., Ferreira A.P. (2020). A New Lipophilic Amino Alcohol, Chemically Similar to Compound FTY720, Attenuates the Pathogenesis of Experimental Autoimmune Encephalomyelitis by PI3K/Akt Pathway Inhibition. Int. Immunopharmacol..

[184] Cui Y.-R., Bu Z.-Q., Yu H.-Y., Yan L.-L., Feng J. (2023). Emodin Attenuates Inflammation and Demyelination in Experimental Autoimmune Encephalomyelitis. Neural Regen. Res..

[185] Ha M.-K., Song Y.H., Jeong S.-J., Lee H.-J., Jung J.H., Kim B., Song H.S., Huh J.-E., Kim S.-H. (2011). Emodin Inhibits Proinflammatory Responses and Inactivates Histone Deacetylase 1 in Hypoxic Rheumatoid Synoviocytes. Biol. Pharm. Bull..

[186] Cheng D.-W., Yue Y.-F., Chen C.-X., Hu Y.-D., Tang Q., Xie M., Liu L., Li D., Zhu H.-L., Cheng M.-L. (2022). Emodin Alleviates Arthritis Pain through Reducing Spinal Inflammation and Oxidative Stress. Mol. Pain..

[187] Hwang J.-K., Noh E.-M., Moon S.-J., Kim J.-M., Kwon K.-B., Park B.-H., You Y.-O., Hwang B.-M., Kim H.-J., Kim B.-S. (2013). Emodin Suppresses Inflammatory Responses and Joint Destruction in Collagen-Induced Arthritic Mice. Rheumatology.

[188] Kshirsagar A.D., Panchal P.V., Harle U.N., Nanda R.K., Shaikh H.M. (2014). Anti-Inflammatory and Antiarthritic Activity of Anthraquinone Derivatives in Rodents. Int. J. Inflam..

[189] Yuan X., Dai B., Yang L., Lin B., Lin E., Pan Y. (2020). Emodin Ameliorates Renal Injury in BXSB Mice by Modulating TNF-α/ICAM-1. Biosci. Rep..

[190] Drory Y., Turetz Y., Hiss Y., Lev B., Fisman E.Z., Pines A., Kramer M.R. (1991). Sudden Unexpected Death in Persons < 40 Years of Age. Am. J. Cardiol..

[191] Cihakova D., Rose N.R. (2008). Chapter 4 Pathogenesis of Myocarditis and Dilated Cardiomyopathy. Advances in Immunology.

[192] Caforio A.L.P., Mahon N.J., Tona F., McKenna W.J. (2002). Circulating Cardiac Autoantibodies in Dilated Cardiomyopathy and Myocarditis: Pathogenetic and Clinical Significance. Eur. J. Heart Fail..

[193] Milenković M., Arsenović-Ranin N., Stojić-Vukanić Z., Bufan B., Vučićević D., Jančić I. (2010). Quercetin Ameliorates Experimental Autoimmune Myocarditis in Rats. J. Pharm. Pharm. Sci..

[194] Okura Y., Yamamoto T., Goto S., Inomata T., Hirono S., Hanawa H., Feng L., Wilson C.B., Kihara I., Izumi T. (1997). Characterization of Cytokine and INOS MRNA Expression in Situ during the Course of Experimental Autoimmune Myocarditis in Rats. J. Mol. Cell Cardiol..

[195] Song Z.-C., Wang Z.-S., Bai J.-H., Li Z., Hu J. (2012). Emodin, a Naturally Occurring Anthraquinone, Ameliorates Experimental Autoimmune Myocarditis in Rats. Tohoku J. Exp. Med..

[196] Zhu W., He X., Cheng K., Zhang L., Chen D., Wang X., Qiu G., Cao X., Weng X. (2019). Ankylosing Spondylitis: Etiology, Pathogenesis, and Treatments. Bone Res..

[197] Lindström U., Olofsson T., Wedrén S., Qirjazo I., Askling J. (2018). Impact of Extra-Articular Spondyloarthritis Manifestations and Comorbidities on Drug Retention of a First TNF-Inhibitor in Ankylosing Spondylitis: A Population-Based Nationwide Study. RMD Open.

[198] Ma C., Wen B., Zhang Q., Shao P.-P., Gu W., Qu K., Shi Y., Wang B. (2019). Emodin Induces Apoptosis and Autophagy of Fibroblasts Obtained from Patient with Ankylosing Spondylitis. Drug Des. Dev. Ther..

[199] Fitsiou E., Pulido T., Campisi J., Alimirah F., Demaria M. (2021). Cellular Senescence and the Senescence-Associated Secretory Phenotype as Drivers of Skin Photoaging. J. Investig. Dermatol..

[200] Callender L.A., Carroll E.C., Beal R.W.J., Chambers E.S., Nourshargh S., Akbar A.N., Henson S.M. (2018). Human CD8+ EMRA T Cells Display a Senescence-Associated Secretory Phenotype Regulated by P38 MAPK. Aging Cell.

[201] Salminen A., Huuskonen J., Ojala J., Kauppinen A., Kaarniranta K., Suuronen T. (2008). Activation of Innate Immunity System during Aging: NF-KB Signaling Is the Molecular Culprit of Inflamm-Aging. Ageing Res. Rev..

[202] Bai D., Ueno L., Vogt P.K. (2009). Akt-Mediated Regulation of NFkappaB and the Essentialness of NFkappaB for the Oncogenicity of PI3K and Akt. Int. J. Cancer.

[203] Kaushik S., Tasset I., Arias E., Pampliega O., Wong E., Martinez-Vicente M., Cuervo A.M. (2021). Autophagy and the Hallmarks of Aging. Ageing Res. Rev..

[204] Zinecker H., Simon A.K. (2022). Autophagy Takes It All—Autophagy Inducers Target Immune Aging. Dis. Model. Mech..

[205] Salminen A., Kaarniranta K., Kauppinen A. (2012). Inflammaging: Disturbed Interplay between Autophagy and Inflammasomes. Aging.

[206] Djavaheri-Mergny M., Amelotti M., Mathieu J., Besançon F., Bauvy C., Souquère S., Pierron G., Codogno P. (2006). NF-KappaB Activation Represses Tumor Necrosis Factor-Alpha-Induced Autophagy. J. Biol. Chem..

[207] Barcellini W., Fattizzo B. (2024). Autoimmune Hemolytic Anemias: Challenges in Diagnosis and Therapy. Transfus. Med. Hemotherapy.

[208] Jiang H., Mao T., Liu Y., Tan X., Sun Z., Cheng Y., Han X., Zhang Y., Wang J., Shi L. (2022). Protective Effects and Mechanisms of Yinchen Linggui Zhugan Decoction in HFD-Induced Nonalcoholic Fatty Liver Disease Rats Based on Network Pharmacology and Experimental Verification. Front. Pharmacol..

[209] Caserta S., Zaccuri A.M., Innao V., Musolino C., Allegra A. (2021). Immune Thrombocytopenia: Options and New Perspectives. Blood Coagul. Fibrinolysis.

[210] Kos M., Tomaka P., Mertowska P., Mertowski S., Wojnicka J., Błażewicz A., Grywalska E., Bojarski K. (2024). The Many Faces of Immune Thrombocytopenia: Mechanisms, Therapies, and Clinical Challenges in Oncological Patients. J. Clin. Med..

[211] Naini M.A., Zargari-Samadnejad A., Mehrvarz S., Tanideh R., Ghorbani M., Dehghanian A., Hasanzarrini M., Banaee F., Koohi-Hosseinabadi O., Tanideh N. (2021). Anti-Inflammatory, Antioxidant, and Healing-Promoting Effects of *Aloe vera* Extract in the Experimental Colitis in Rats. Evid.-Based Complement. Alternat. Med..

[212] Liu L., Zhang Y., Tang X.-R., Jia G.-B., Zhou S., Yue G.-L., He C.-S. (2024). Effect of Emodin on Acute Lung Injury: A Meta-Analysis of Preclinical Trials. BMC Pulm. Med..

[213] Gao H., Ren Y., Liu C. (2022). Aloe-Emodin Suppresses Oxidative Stress and Inflammation via a PI3K-Dependent Mechanism in a Murine Model of Sepsis. Evid.-Based Complement. Alternat Med..

[214] Cui J., Wang S., Bi S., Zhou H., Sun L. (2024). Emodin-Based Regulation and Control of Serum Complement C5a, Oxidative Stress, and Inflammatory Responses in Rats with Urosepsis via AMPK/SIRT1. Iran. J. Allergy Asthma Immunol..

